# Artemisinin exerts a protective effect in the MPTP mouse model of Parkinson's disease by inhibiting microglial activation via the TLR4/Myd88/NF‐KB pathway

**DOI:** 10.1111/cns.14063

**Published:** 2023-01-24

**Authors:** Jing Lv, Jing Zhu, Peihan Wang, Tongyu Liu, Jiang Yuan, Huan Yin, Yiran Lan, Qiang Sun, Zhifeng Zhang, Guoda Ding, Chenxi Zhou, Huajie Wang, Zihan Wang, Yunfu Wang

**Affiliations:** ^1^ Department of Neurology Graduate Training Base of Jinzhou Medical University, Affiliated Hospital of Hubei Medical College, Taihe Hospital Shiyan China; ^2^ Institute of Neuroscience Hubei University of Medicine Shiyan China; ^3^ Department of Neurology Taihe Hospital of Hubei University of Medicine Shiyan China; ^4^ Sinopharm Dongfeng General Hospital, Hubei University of Medicine Shiyan China

**Keywords:** artemisinin, inflammation, microglia, Parkinson's disease, toll‐like receptor 4

## Abstract

**Aims:**

We performed cell and animal experiments to explore the therapeutic effect of artemisinin on Parkinson's disease (PD) and the TLR4/Myd88 signaling pathway.

**Methods:**

C57 mice were randomly divided into the blank, 1‐methyl‐4‐phenyl‐1,2,3,6‐tetrahydropyridine (MPTP)‐induced and artemisinin‐treated groups. Clinical symptoms, the number of dopaminergic (DAergic) neurons in the substantia nigra, and microglial cell activation were compared among the three groups. Subsequently, BV‐2 cell activation and TLR4/Myd88 pathway component expression were compared among the blank, MPP^+^‐treated, artemisinin‐treated, and TLR4 activator‐treated groups.

**Results:**

Behavioral symptoms were improved, the number of DAergic neurons in the substantia nigra of the midbrain was increased, and microglial cell activation was decreased in artemisinin‐treated MPTP‐induced PD model mice compared with control‐treated MPTP‐induced PD model mice (*p* < 0.05). The cell experiments revealed that artemisinin treatment reduced MPP^+^‐induced BV‐2 cell activation and inhibited the TLR4/Myd88 signaling pathway. Moreover, the effect of artemisinin on the BV‐2 cell model was inhibited by the TLR4 activator LPS (*p* < 0.05).

**Conclusion:**

Artemisinin may reduce damage to DAergic neurons in a PD mouse model by decreasing microglial activation through the TLR4‐mediated MyD88‐dependent signaling pathway. However, this finding cannot explain the relationship between microglia and DAergic neurons.

## Graphical abstract

At present, the drug treatment of Parkinson's disease cannot improve the damage of dopaminergic neurons. The protective mechanism of artemisinin which is economical and safe to protect dopaminergic neurons is discussed.

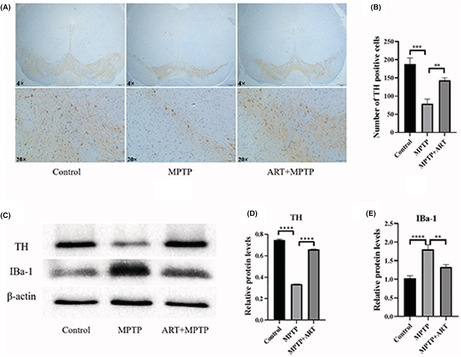

## 1 BACKGROUND

Parkinson's disease (PD) is a neurodegenerative disease mainly characterized by extrapyramidal symptoms. It affects >1% of individuals ≥65 years old, and its prevalence is expected to double by 2030.^1^ Over the course of the disease, clinical symptoms worsen, and patient quality of life declines. Thus, PD imposes an increasing economic and social burden on families and society. A decrease in the number of dopaminergic (DAergic) neurons in the substantia nigra pars compacta (SNpc), which is the main pathological basis of PD, disrupts the balance between dopamine (DA) and acetylcholine in the body, resulting in the clinical symptoms of PD. Abnormal aggregation of microglia in the substantia nigra of the midbrain has been observed in humans and animals.^2^, ^3^ Activation of microglial cells is related to damage to DAergic neurons.^4^

Microglia are the main immune cells in the central nervous system (CNS), accounting for 10% of all glial cells in the CNS. Most of them are distributed in the midbrain, and they maintain the homeostasis of the neuronal microenvironment. Usually, microglia are in an inactive state. When the neuronal microenvironment changes, they are activated by stimuli and release inflammatory factors such as TNF‐α and IL‐1β, which damage neurons. Many studies have shown that microglial cells in the brain are activated before the CNS is damaged.^5^ TLR4, which is widely expressed on immune cell membranes, is highly expressed on the microglial cell membrane in the CNS and plays a major role in the neuroinflammatory response to CNS injury. The expression of TLR4 is upregulated in the 1‐methyl‐4‐phenyl‐1,2,3,6‐tetrahydropyridine (MPTP) mouse model of PD. In contrast, TLR4 deficiency has a protective effect on mice.^6^, ^7^, ^8^ Therefore, TLR4 may mediate the release of inflammatory factors by microglia, thereby damaging DAergic neurons in the SNpc.

The main drugs currently used to treat PD include DA supplements, DA receptor agonists, catechol‐O‐methyltransferase inhibitors, and monoamine oxidase inhibitors.^9^ These drugs, which can increase the content of DA in the body or reduce the metabolism of DA, can temporarily relieve clinical symptoms but cannot reduce damage to DAergic neurons. The therapeutic effect of these treatments declines over time, and these drugs have many side effects. Therefore, an effective strategy to treat PD by reducing the number of injured DAergic neurons is needed.

Artemisinin is extracted from the flowers and leaves of the Chinese herb Artemisia annua. It is well known that artemisinin is efficacious in the treatment of malaria. As our understanding of the effects of artemisinin has increased, we have found that artemisinin not only has antimalarial effects but also has other beneficial effects, such as antioxidant^10^ and antitumor^11^ effects. More importantly, some studies have shown that artemisinin derivatives have neuroprotective effects,^12^, ^13^ but their functional mechanisms are different. In particular, there have been few studies on the effect of artemisinin in PD, and the therapeutic effect and potential mechanism of artemisinin in PD remain unclear.

A common PD animal model^14^ was established by intraperitoneal injection of MPTP into C57 mice. Animal experiments have confirmed that artemisinin has a therapeutic effect on PD model mice, and this effect is related to microglial activation. Upon administration, MPTP can penetrate the blood–brain barrier in mice and be converted to MPP^+^ through a series of reactions. Treatment of BV‐2 cells with MPP^+^ can simulate the effect of MPP^+^ on microglial cells after passing through the blood–brain barrier. Cell experiments have shown that MPP^+^ can activate microglia, and animal experiments have indicated that artemisinin can protect DA and their interactions with TLR4/Myd88/NF‐KB pathway components. Through animal experiments and cell experiments, we aimed to clarify the protective effect of artemisinin on dopaminergic neurons in Parkinson's disease model rats and the underlying mechanism.

## 2 MATERIALS AND METHODS

### 2.1 Animal model establishment

Male C57 mice (20–25 g, aged 8 weeks) were purchased from the Animal Research Center of Hubei Medical University (Shiyan, China). All animals were housed on a 12‐h light/dark cycle at a temperature of 22 ± 2°C. The animals had free access to standard diet and water. All animal experiments were carried out in compliance with the Guide for the Care and Use of Laboratory Animals established by the National Institutes of Health and were approved by the Animal Care Committee of the Peking Union Medical College and Chinese Academy of Medical Sciences. The animal experiment is described above. All mice were randomly divided into 3 groups (the control, model, and artemisinin treatment groups). To construct the animal model, mice were intraperitoneally injected with MPTP (25 mg/kg, dissolved in 0.9% normal saline, Sigma, m0896) once a day for 7 consecutive days. The mice in the artemisinin treatment group were intraperitoneally injected with artemisinin (18 mg/kg, dissolved in DMSO, Shyuanye, S31425) 2 h after MPTP injection once a day for 14 consecutive days. An equal volume of normal saline (Wuhanfuxing, H20043320) was intraperitoneally injected into the control mice once a day. 
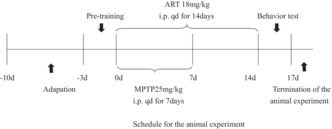


### 2.2 Behavioral experiments

**FIGURE 1:**
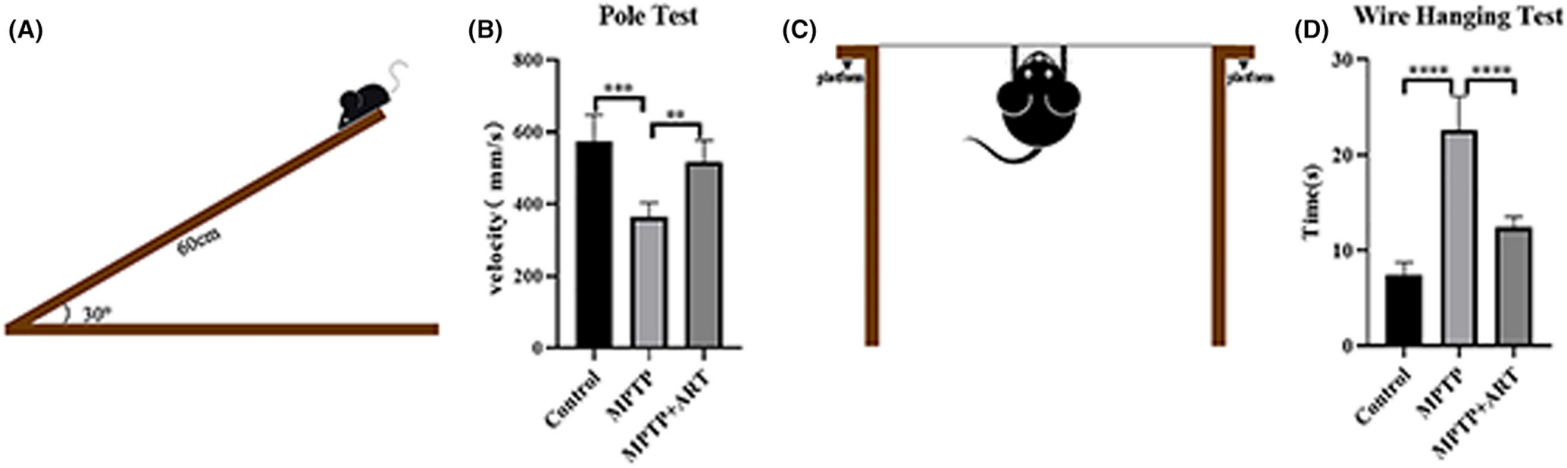
Artemisinin attenuated MPTP‐induced motor impairment. (A, C) Graphs of the pole test and wire hanging test. (B) Results of the pole test. (D) Results of the wire hanging test. *n* = 5, ****p < 0.0001, ***p < 0.001, **p < 0.01.

The pole test was used to evaluate the degree of motor dysfunction. The two devices used are shown in Figure 1A,C. The mice were placed at the top of the pole and allowed to climb down it. The time required to climb down the pole was recorded. The wire hanging test was used to assess the ability to overcome challenges and the muscle strength of the mice. The forelegs of each mouse were placed in the middle of a wire, and the time required to reach one of the square platforms was recorded.

### 2.3 Immunohistochemistry (IHC) Inhalation of isopentane for anesthesia Animals are anesthetized

Animals were anesthetized by inhalation of isoflurane. The mice were decapitated after the behavioral experiments. Midbrain tissue was isolated, fixed in 4% paraformaldehyde and embedded in paraffin 48 h later. Paraffin sections (5 μm thick) were cut for IHC. The paraffin sections were dewaxed, rehydrated, incubated with a TH antibody (1:500, Servicebio, GB11181) followed by HRP‐labeled goat anti‐rabbit IgG (H+L) (1:50, Beyotime, A0208), stained with DAB (ZSGB‐Bio, ZLI‐9018), and sealed with neutral balsam. The sections were observed with an Olympus BA51 photomicroscope (Tokyo, Japan). ImageJ 1.53 E software was used to count the stained cells.

### 2.4 Western blot (WB) analysis

RIPA lysis buffer (Applygen, C1053) was mixed with a phosphatase inhibitor cocktail (1:50, Beyotime, P1081) and phenylmethanesulfonyl fluoride (1:100, dissolved in DMSO, Roche, 10,837,091,001). Midbrain tissues and BV‐2 cells were lysed in RIPA lysis buffer, and the supernatant, which contained protein, was collected. After electrophoresis and transfer, the membranes (0.2 μm, Millipore) were blocked and incubated with TH (1:1000, CST, 13106S), TLR4 (1:1000, CST, 14358S), Myd88 (1:1000, CST, 4283S), NF‐KB (1:1000, CST, 8242T), p‐NF‐KB (1:1000, CST, 3033T) and β‐actin (1:1000, CST, 8457S) antibodies overnight at 4°C. After being washed, the membranes were incubated with HRP‐labeled goat anti‐rabbit IgG (H+L) (1:5000, Beyotime, A0208) for 2 h at RT. After the membranes were washed again, the protein bands were detected by chemiluminescent HRP substrate (Millipore, P90720) and analyzed with ImageJ 1.53E software.

### 2.5 Cell culture and treatment

BV‐2 cells were purchased from Shanghai Zhong Qiao Xin Zhou and cultured in Dulbecco's modified Eagle's medium (DMEM, AG29853165, HyClone) containing 10% FBS (Vivacell, C04001‐500) and 1% penicillin–streptomycin (Beyotime, C0222) in a humidified atmosphere containing 5% CO_2_ at 37°C in an incubator. For artemisinin stimulation, BV‐2 cells in 96‐well cell culture plates were treated with the indicated concentration of artemisinin. BV‐2 cells from the same 60 mm cell culture dish were divided into 4 groups (the control, PD cell model, artemisinin treatment, and TLR4 activator treatment groups). To establish the PD cell model, BV‐2 cells were incubated with 1 μM MPP^+^ for 24 h at 37°C. BV‐2 cells in the artemisinin treatment group were incubated with 1 μM MPP^+^ (dissolved in DMEM, MCE, HY‐W008719) for 24 h after treatment with 5 μM artemisinin for 2 h. BV‐2 cells in the TLR4 activator treatment group were incubated with 1 μM MPP^+^ and 10 μg/L LPS (Beyotime, S1732) for 24 h after treatment with 5 μM artemisinin for 2 h in an incubator.

### 2.6 Measurement of TNF‐α and IL‐1β levels

After the indicated treatments, the cell supernatant of each group was collected in a 1.5 ml EP tube, and the production of TNF‐α and IL‐1β was measured. Commercially available ELISA kits for TNF‐α (Neobioscience, EMC102a) and IL‐1β (Neobioscience, EMC001b) were utilized to quantify the levels of these cytokines according to the manufacturer's instructions. The optical density at 450 nm was measured by using a multifunctional enzyme labelling instrument (SpectraMax i3, Afghanistan).

### 2.7 Flow cytometry analysis

BV‐2 cells from the same 60 mm cell culture dish were divided into 4 groups. After the cells were treated as described above, they were harvested with 0.25% trypsin (Beyotime, C0207) to prepare single‐cell suspensions. BV‐2 cell apoptosis was assessed using an Annexin V‐FITC/PI apoptosis detection kit (Lablead, AF2020‐50T) according to the manufacturer's instructions. The cells were analyzed with a flow cytometer (Cytoflex/Beckman Coulter, China).

### 2.8 Cell viability

Cell viability measurement was performed using the CCK‐8 assay (Biosharp, BS350B) according to the instruction manual. The optical density at 450 nm was measured by using a multifunctional enzyme labelling instrument (SpectraMax i3, Afghanistan).

### 2.9 Immunofluorescence

Cells in 24‐well plates were prepared for immunofluorescence as described in Section 2.5. After the cells were treated as described above, they underwent fixation in 4% paraformaldehyde, permeabilization, blocking, Iba‐1 antibody (1:500, Abcam, ab178846) incubation, rinsing, APC‐conjugated goat anti‐rabbit IgG H&L antibody (1:100, Bioss, bs‐0295G‐APC) incubation, rinsing, DAPI staining, and rinsing again. The cells were observed with an inverted fluorescence microscope (Leica dmi6000B, Germany), and ImageJ 1.53E software was used to count the stained cells.

### 2.10 Statistical analysis

All the data, which are presented as the mean ± standard deviation (SD), conformed to a normal distribution. Comparisons between groups were performed using paired one‐way analysis of variance (ANOVA) followed by the least significant difference test for pairwise comparisons. All data analyses were performed using GraphPad Prism 8.0. A two‐tailed *p* value <0.05 was considered statistically significant.

## 3 RESULTS

### 3.1 Artemisinin attenuates MPTP‐induced motor impairment

To assess the ability of artemisinin to alleviate the clinical symptoms of PD model mice, three groups of mice were subjected to the pole test and wire hanging test. Motor dysfunction negatively affects the daily lives of patients with PD and is key for PD diagnosis according to the latest clinical practice guidelines.^9^ In the pole test (Figure 1A,B), the velocity of mice in the MPTP‐treated group was slower than that of mice in the blank group (572.8 ± 74.61 mm/s (Control) vs. 365.1 ± 38.94 mm/s (MPTP), *p* = 0.0004); however, velocity was restored to normal levels after treatment with artemisinin (516.9 ± 60.15 mm/s (Artemisinin) vs. 365.1 ± 38.94 mm/s (MPTP), *p* = 0.0045). In the wire hanging test (Figure 1C,D), which was used to assess the muscle strength of the mice and their ability to overcome challenges, mice in the model group required a longer time to reach the platform than mice in the blank control group (7.4 ± 1.342 s (Control) vs. 22.6 ± 3.507 s (MPTP), *p* < 0.0001). And the artemisinin‐treated mice performed better than the MPTP‐treated mice (12.4 ± 1.14 s (Artemisinin) vs. 22.6 ± 3.507 s (MPTP), *p* < 00001).

### 3.2 The ability of artemisinin to attenuate DAergic neuron injury in PD model mice may be related to the inhibition of microglial activation

**FIGURE 2:**
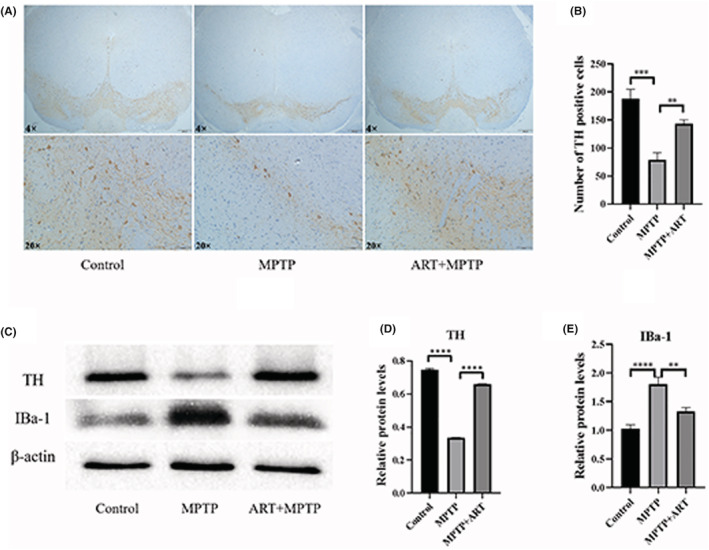
The ability of artemisinin to attenuate DAergic neuron injury in PD model mice may be related to the inhibition of microglial activation. (A) Immunohistochemical staining of TH in the SNpc. (B) The statistical results showed that there were fewer TH^+^ cells in the SNpc in the model group than in the blank group, and more of these cells in the artemisinin treatment group than in the model group. (C) WB analysis of TH and Iba‐1 in the midbrain. (D) The statistical results showed that the relative TH level in the SNpc was lower in the model group than in the blank group and higher in the artemisinin treatment group than in the model group. (E) The statistical results showed that the relative IBa‐1 level in the midbrain was higher in the model group than in the blank group and lower in the artemisinin treatment group than in the model group. *n* = 3, **p < 0.01, ****p < 0.0001, ***p < 0.001.

The key pathological feature of PD is a decrease in the number of DAergic neurons in the SNpc. Tyrosine hydroxylase (TH), which is expressed in DAergic neurons, is an enzyme that converts the amino acid L‐tyrosine into dihydroxyphenylalanine (DOPA) and is also a rate‐limiting enzyme for the synthesis of levodopa. Therefore, TH^+^ cells are DAergic neurons. IHC of midbrain tissues (Figure 2A,B) showed that the number of TH^+^ cells was decreased in the MPTP group (188 ± 16.59 (Control) vs. 79.25 ± 12.58 (MPTP), *p* < 00001), indicating successful modeling, and increased after artemisinin treatment (143 ± 7.528 (Artemisinin) vs. 79.25 ± 12.58 (MPTP), *p* = 00002), indicating that the drug successfully ameliorated PD pathology. The WB results (Figure 2C,D) revealed the same findings (0.746 ± 0.00842 (Control) vs. 0.335 ± 0.00147 (MPTP), *p* < 00001; 0.66 ± 0.00157 (Artemisinin) vs. (MPTP), *p* < 00001). Surprisingly, the expression of IBa‐1, a cytoskeletal protein expressed by microglia, in the midbrain was increased in all three groups when microglia were activated (Figure 2C,E). The IBa‐1 content in the midbrain showed a greater increase in model mice (1.025 ± 0.0728 (Control) vs. 1.808 ± 0.12 (MPTP), *p* < 00001) and decreased after artemisinin treatment (1.331 ± 0.00681 (Artemisinin) vs. 1.808 ± 0.12 (MPTP), *p* = 0.0015).

### 3.3 Artemisinin has an inhibitory effect on microglial cell activation induced by MPP^+^

**FIGURE 3:**
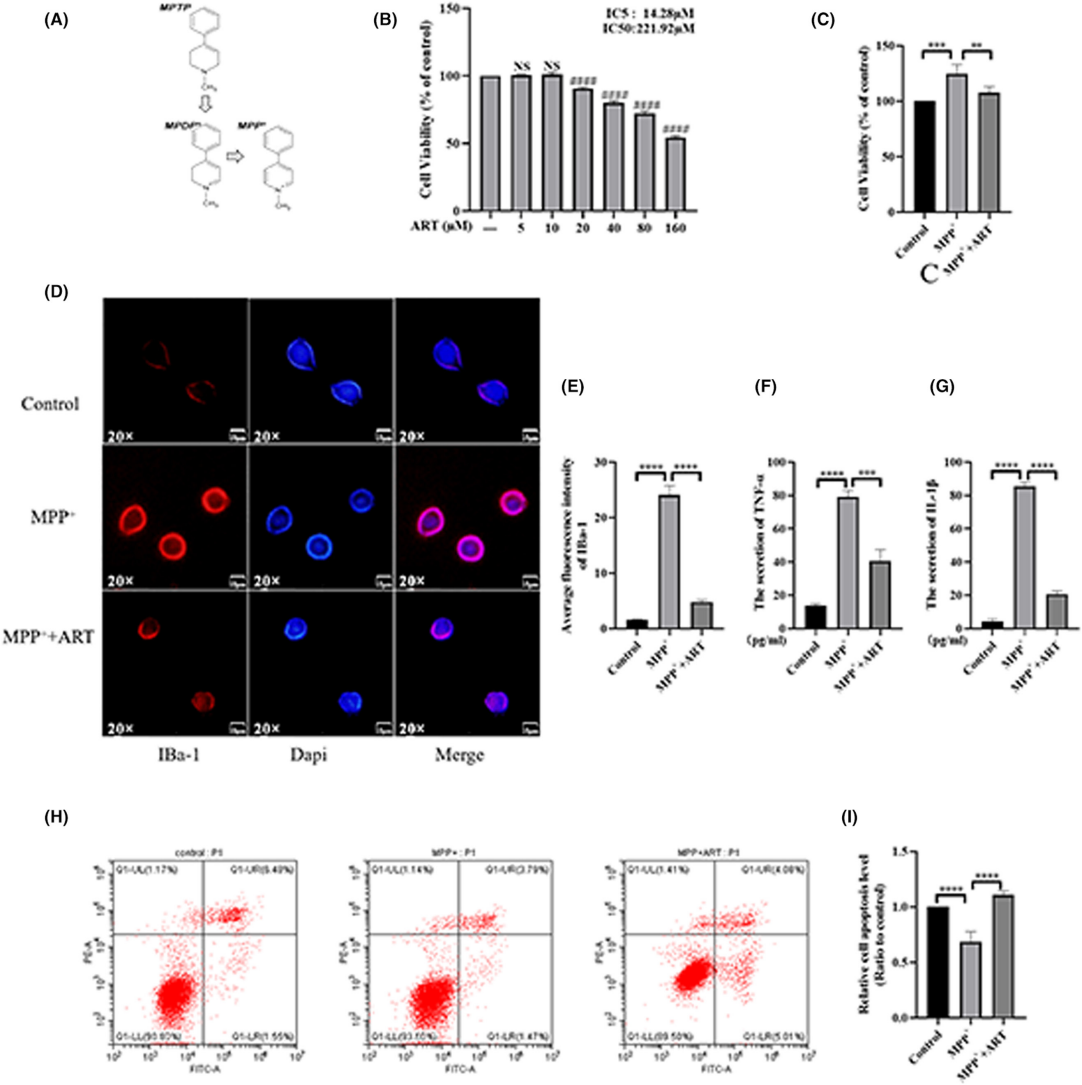
Artemisinin has an inhibitory effect on microglial cell activation induced by MPP^+^. (A) Schematic of the MPP^+^ conversion process. (B) Cell viability following treatment with 0, 5, 10, 20, 40, and 80 M artemisinin was 100%, 100.8% ± 0.212%, 101.2% ± 1.69%, 90.75% ± 0.854%, 80.22% ± 1.345%, 72.21% ± 1.733%, and 54.34% ± 1.335%, respectively, as determined by the CCK‐8 assay. *n* = 5, ^####^ *p* < 0.0001 vs. Control, ns = *p* > 0.05. Cell viability after treatment with 5 and 10 M artemisinin was not significantly different from that in the absence of artemisinin. Cell viability was decreased after treatment with artemisinin in the range of 20–160 M. (C) The CCK‐8 assay showed that cell activity was increased in the MPP^+^ group and decreased after artemisinin treatment. (D) Fluorescence showing the expression level of IBa‐1 in each group. (E) The expression of IBa‐1 was increased in the MPP^+^ group and decreased after artemisinin treatment. (F, G) ELISA was used to measure the content of TNF‐α and IL‐1β in the cell supernatant from the three groups, and the results indicated that TNFα and IL‐1β levels were increased after MPP^+^ treatment and decreased after artemisinin treatment. (H) The number of apoptotic cells in 10,000 cells in each group was determined by flow cytometry. (I) Statistical analysis showed that there were fewer apoptotic cells in the MPP^+^ group than in the blank group and that the proportion of apoptotic cells was increased after artemisinin treatment. *n* = 3, ****p < 0.0001, ***p < 0.001, **p < 0.01.

After passing through the blood–brain barrier, liposoluble MPTP was gradually converted to water‐soluble MPP^+^ after a series of metabolic processes (Figure 3A). Microglia, which monitor the microenvironment in the brain, are activated before neuronal damage in the brain.^15^ Therefore, MPP^+^‐treated BV‐2 cells are the best cell model for simulating the changes in microglia in the brain after intraperitoneal injection of MPTP. BV‐2 microglial cells isolated from C57 mice are usually in a resting state. When the surroundings change, microglia become activated. Compared with resting microglia, activated microglia exhibit increased cell activity, Iba‐1 expression and secretion of inflammatory factors and decreased apoptosis. Cell viability was measured as the ratio of the number of cells per unit time to the initial total number of cells. Apoptosis is inevitable during the growth of any cell. Flow cytometry analysis was used to determine the proportion of apoptotic cells relative to total cells.

**FIGURE 4:**
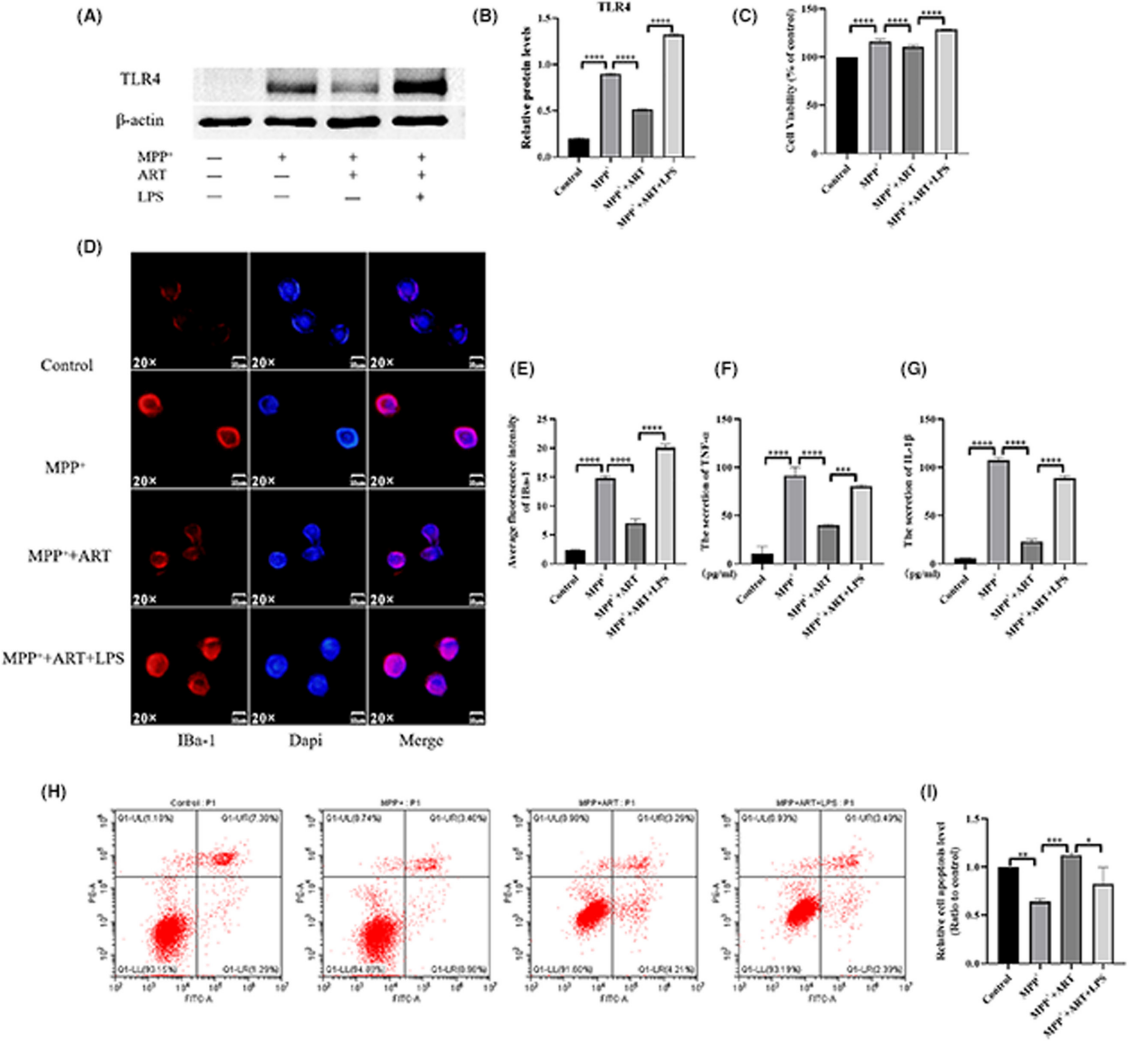
Artemisinin attenuates cell activation by inhibiting TLR4 on the cell membrane. (A) WB results showing the expression of TLR4 in the four groups. (B) WB results showing that the expression of TLR4 was increased in the MPP^+^ group compared with the blank group (0.2001 ± 0.0098 (Control) vs. 0.881 ± 0.0083 (MPP^+^), ^****^ *p* < 0.0001) and decreased after artemisinin treatment (0.514 ± 0.0094 (Artemisinin) vs. 0.881 ± 0.0083 (MPP^+^), ^****^ *p* < 0.0001). LPS was administered after artemisinin treatment, and the expression of TLR4 further increased (*n* = 3, ^****^ *p* < 0.0001). (C) CCK‐8 assay results showing that cell survival was increased in the MPP^+^ group compared with the blank group (100% (Control) vs. 116.1 ± 2.54% (MPP^+^), ^****^ *p* < 0.0001, *n* = 5) and decreased after artemisinin treatment (110.6 ± 2.083% (Artemisinin) vs. 116.1 ± 2.54% (MPP^+^), ^****^ *p* < 0.0001, *n* = 5). After adding LPS, the expression of TLR4 was further increased (128.6 ± 0.516% (LPS) vs. (110.6 ± 2.083% (Artemisinin), ^****^ *p* < 0.0001, *n* = 5). (D) Fluorescence showing the expression level of IBa‐1 in each group. (E) The relative fluorescence intensity of IBa‐1 was increased in the MPP^+^ group (2.434 ± 0.0753 (Control) vs. 14.78 ± 0.447 (MPP^+^), ^****^ *p* < 0.0001, *n* = 3) and decreased after artemisinin treatment (7.016 ± 0.783 (Artemisinin) vs. 14.78 ± 0.447 (MPP^+^), ^****^ *p* < 0.0001, *n* = 3). LPS was administered following artemisinin treatment, and the relative fluorescence intensity of IBa‐1 further increased (20.08 ± 0.6002 (LPS) vs. 7.016 ± 0.783 (Artemisinin), ^****^ *p* < 0.0001, *n* = 3). (F) The levels of TNF‐α in the supernatant in the MPP^+^ group were higher (1.995 ± 0.2825 (Control) vs. 80.17 ± 4.762 (MPP^+^), ^****^ *p* < 0.0001, *n* = 3) and decreased after artemisinin treatment (4.43 ± 7.744 (Artemisinin) vs. 80.17 ± 4.762 (MPP^+^), ***p = 0.0002 < 0.001, *n* = 3). LPS was administered following artemisinin treatment, and the level of TNF‐α further increased (93.86 ± 5.783 (LPS) vs. 44.43 ± 7.744 (Artemisinin), ^****^ *p* < 0.0001, *n* = 3). (G) The levels of IL‐1β in the supernatant in the MPP^+^ group were higher (5.63 ± 0.3979 (Control) vs. 107.3 ± 2.926 (MPP^+^), ^****^ *p* < 0.0001, *n* = 3) and decreased after artemisinin treatment (23.02 ± 2.264 (Artemisinin) vs. 107.3 ± 2.926 (MPP^+^), ^****^ *p* < 0.0001, *n* = 3). LPS was administered following artemisinin treatment, and the level of TNF‐α further increased (88.9 ± 2.655 (LPS) vs. 23.02 ± 2.264 (Artemisinin), ^****^ *p* < 0.0001, *n* = 3). (H) The number of apoptotic cells in 10,000 cells in each group was determined by flow cytometry. (I) Statistical analysis showed that the proportion of apoptotic cells in the MPP^+^ group was lower than that in the blank group (1 (Control) vs. 0.6418 ± 0.2979 (MPP^+^), **p = 0.0046 < 0.01, *n* = 3) and that the proportion of apoptotic cells was increased after artemisinin treatment (1.124 ± 0.02035 (Artemisinin) vs. 0.6418 ± 0.2979 (MPP^+^), ***p = 0.0007 < 0.001, *n* = 3). LPS was administered following artemisinin treatment, and the proportion of apoptotic cells further increased (0.8245 ± 0.1714 (LPS) vs. 1.124 ± 0.02035 (Artemisinin), *p = 0.013 < 0.05, *n* = 3).

The viability of ART‐treated cells was also evaluated, and the results (Figure 3B) showed that the IC50 of artemisinin was 221.92 μM and the IC5, which is usually used as the upper dose limit, was 14.28 μM. Moreover, there was no significant difference in cell viability between the 5 μM ART‐treated group (100.8% ± 0.212% vs. 100% (Control), *p* = 0.816) and 10 μM ART‐treated group (101.2% ± 1.69% vs. 100% (Control), *p* = 0.477). Therefore, we chose 5 μM artemisinin for the experiment. The CCK‐8 assay results (Figure 3C) showed that cell activity was increased in the MPP^+^ group (100% (Control) vs. 124.5% ± 8.673% (MPP^+^), *p* = 0.0006) and decreased after artemisinin treatment (107.8% ± 5.463% (Artemisinin) vs. 124.5% ± 8.673% (MPP^+^), *p* = 0.0079). Immunofluorescence (Figure 3D,E) revealed that IBa‐1 expression was higher in the MPP^+^ group than in the control group (1.59 ± 0.128 (Control) vs. 24.08 ± 1.691 (MPP^+^), *p* < 0.0001) and decreased after artemisinin treatment (4.785 ± 0.384 (Artemisinin) vs. 24.08 ± 1.691 (MPP^+^), *p* < 0.0001). ELISA (Figure 3F,G) was used to measure the content of TNF‐α and IL‐1β in the cell supernatant from the three groups, and the results showed that TNF‐α and IL‐1β levels increased after MPP^+^ treatment (13.6 ± 1.444 (Control) vs. 79.02 ± 3.846 (MPP^+^), *p* < 0.0001; 4.3 ± 1.711 (Control) vs. 85.4 ± 2.446 (MPP^+^), *p* < 0.0001) and decreased after artemisinin treatment (40.58 ± 6.839 (Artemisinin) vs. 79.02 ± 3.846 (MPP^+^), *p* = 0.0001; 20.64 ± 2.07 (Artemisinin) vs. 85.4 ± 2.446 (MPP^+^), *p* < 0.0001). Flow cytometry was used to determine the proportion of early apoptotic and late apoptotic cells per 10,000 cells in the three groups (Figure 4H,I). The results indicated that MPP^+^ reduced the cell apoptosis rate (1 (Control) vs. 0.6859 ± 0.0915 (MPP^+^), *p* < 0.0001) and that this effect was reversed by artemisinin treatment (1.109 ± 0.0343 (Artemisinin) vs. 0.686 ± 0.0915 (MPP^+^), *p* < 0.0001). Therefore, artemisinin can inhibit the activation of BV‐2 cells induced by MPP^+^.

### 3.4 Artemisinin attenuates cell activation by inhibiting TLR4 on the microglial cell membrane

WB (Figure 4A,B) analysis revealed that the expression of TLR4 in the three groups was different; therefore, the TLR4 activator LPS was adding on base of artemisinin treatment. After adding LPS, the results showed that TLR4 expression further increased (0.514 ± 0.00944 (Artemisinin) vs. 1.323 ± 0.0102 (LPS), *p* < 0.0001). LPS inhibits the effect of artemisinin. The results of the CCK‐8 assay (Figure 4C), immunofluorescence (Figure 4D,E), ELISA (Figure 4F,G), and flow cytometry (Figure 4H,I) confirmed these findings. Therefore, we concluded that artemisinin attenuates cell activation by inhibiting TLR4 on the microglial cell membrane.

### 3.5 Artemisinin attenuates cell activation via the TLR4/Myd88/NF‐KB pathway in BV‐2 cells injured by MPP^+^

**FIGURE 5:**
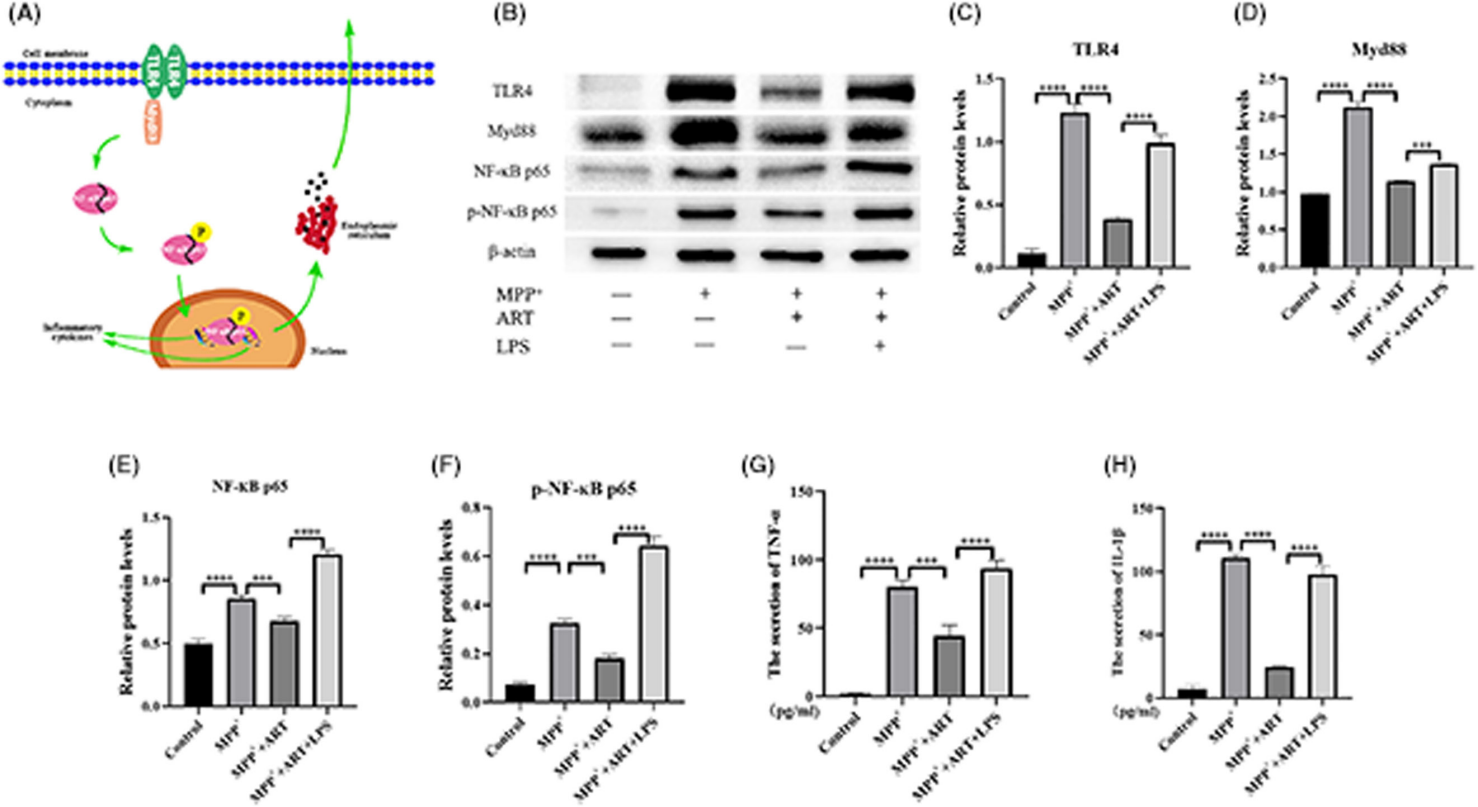
Artemisinin attenuates cell activation via the TLR4/Myd88/NF‐ĸB pathway in BV‐2 cells injured by MPP^+^. (A) Schematic of the TLR4/Myd88/NF‐ĸB pathway. (B) WB results showing the expression of TLR4/Myd88/NF‐ĸB pathway components in the four groups. (C) WB results showing that the expression of TLR4 was increased in the MPP^+^ group compared with the blank group and decreased after artemisinin treatment. LPS was administered following artemisinin treatment, and TLR4 expression further increased. (D) WB results showing that the expression of Myd88 was increased in the MPP^+^ group compared with the blank group and decreased after artemisinin treatment. LPS was administered following artemisinin treatment, and Myd88 expression further increased. (E) WB results showing that the expression of NF‐ĸB p65 was increased in the MPP^+^ group compared with the blank group and decreased after artemisinin treatment. LPS was administered following artemisinin treatment, and NF‐ĸB p65 expression further increased. (F) WB results showing that the expression of p‐NF‐ĸB p65 was increased in the MPP^+^ group compared with the blank group and decreased after artemisinin treatment. LPS was administered following artemisinin treatment, and p‐NF‐ĸB p65 expression further increased. (G) ELISA was used to measure the content of TNF‐α in the cell supernatant in the 4 groups, and the results indicated that TNF‐α levels increased (10.8 ± 7.418 (Control) vs. 91.47 ± 8.867 (MPP^+^), ****p < 0.0001) after MPP^+^ treatment and decreased after artemisinin treatment (40.08 ± 1.099 (Artemisinin) vs. 91.47 ± 8.867 (MPP^+^), ****p < 0.0001). LPS was administered following artemisinin treatment, and TNF‐α expression further increased (80.3 ± 1.72 (LPS) vs. 40.08 ± 1.099 (Artemisinin), ***p = 0.0001 < 0.001). (H) ELISA was used to measure the content of IL‐1β in the cell supernatant in the 4 groups, and the results indicated that TNF‐α levels increased (7.043 ± 2.97 (Control) vs. 110.9 ± 1.886 (MPP^+^), ****p < 0.0001) after MPP^+^ treatment and decreased after artemisinin treatment (24.62 ± 0.481 (Artemisinin) vs. 110.9 ± 1.886 (MPP^+^), ****p < 0.0001). LPS was administered following artemisinin treatment, and IL‐1β expression further increased (97.57 ± 6.919 (LPS) vs. 24.62 ± 0.481 (Artemisinin), ****p < 0.0001).

After the expression of TLR4 on the microglial cell membrane was upregulated, the expression levels of the downstream proteins Myd88, NF‐KB and p‐NF‐KB were increased, and the release of inflammatory factors, such as TNF‐α and IL‐β, was increased (Figure 5A). The results of WB (Figure 5B–F) were that TLR4, Myd88, NF‐ĸB, and p‐NF‐ĸB were increased in the MPP^+^ group compared with the blank group (0.1155 ± 0.0355 (Control) vs. 1.23 ± 0.072 (MPP^+^), ****p < 0.0001; 0.9763 ± 0.00111 (Control) vs. 2.12 ± 0.0783 (MPP^+^), ****p < 0.0001; 0.4989 ± 0.0366 (Control) vs. 0.855 ± 0.028 (MPP^+^), ****p < 0.0001; 0.0737 ± 0.008696 (Control) vs. 0.3274 ± 0.0144 (MPP^+^), ****p < 0.0001), decreased after artemisinin treatment (0.3839 ± 0.02454 (Artemisinin) vs. 1.23 ± 0.072 (MPP^+^), ****p < 0.0001; 1.145 ± 0.004908 (Artemisinin) vs. 2.12 ± 0.0783 (MPP^+^), ****p < 0.0001; 0.6764 ± 0.02988 (Artemisinin) vs. 0.855 ± 0.028 (MPP^+^), ***p = 0.0008; 0.1804 ± 0.01625 (Artemisinin) vs. 0.3274 ± 0.0144 (MPP^+^), ***p = 0.0002) and increased after LPS added on the basis of artemisinin treatment (0.9879 ± 0.06892 (LPS) vs. 0.3839 ± 0.02454 (Artemisinin), ****p < 0.0001; 1.369 ± 0.004091 (LPS) vs. 1.145 ± 0.004908 (Artemisinin), ***p = 0.0005; 1.203 ± 0.03856 (LPS) vs. 0.6764 ± 0.02988 (Artemisinin), ****p < 0.0001; 0.6443 ± 0.03967 (LPS) vs. 0.1804 ± 0.01625 (Artemisinin), ****p < 0.0001). The ELISA results of TNF‐α and IL‐1β were the same trend (Figure 5G,H). Inflammatory factors have detrimental effects on neuronal cells, including DAergic neurons. Therefore, we concluded that artemisinin reduces MPP^+^‐induced activation of BV‐2 cells by inhibiting TLR4/Myd88/NF‐KB signaling.

### 3.6 Changes in the TLR4/Myd88/NF‐KB pathway in artemisinin‐treated PD model mice

**FIGURE 6:**
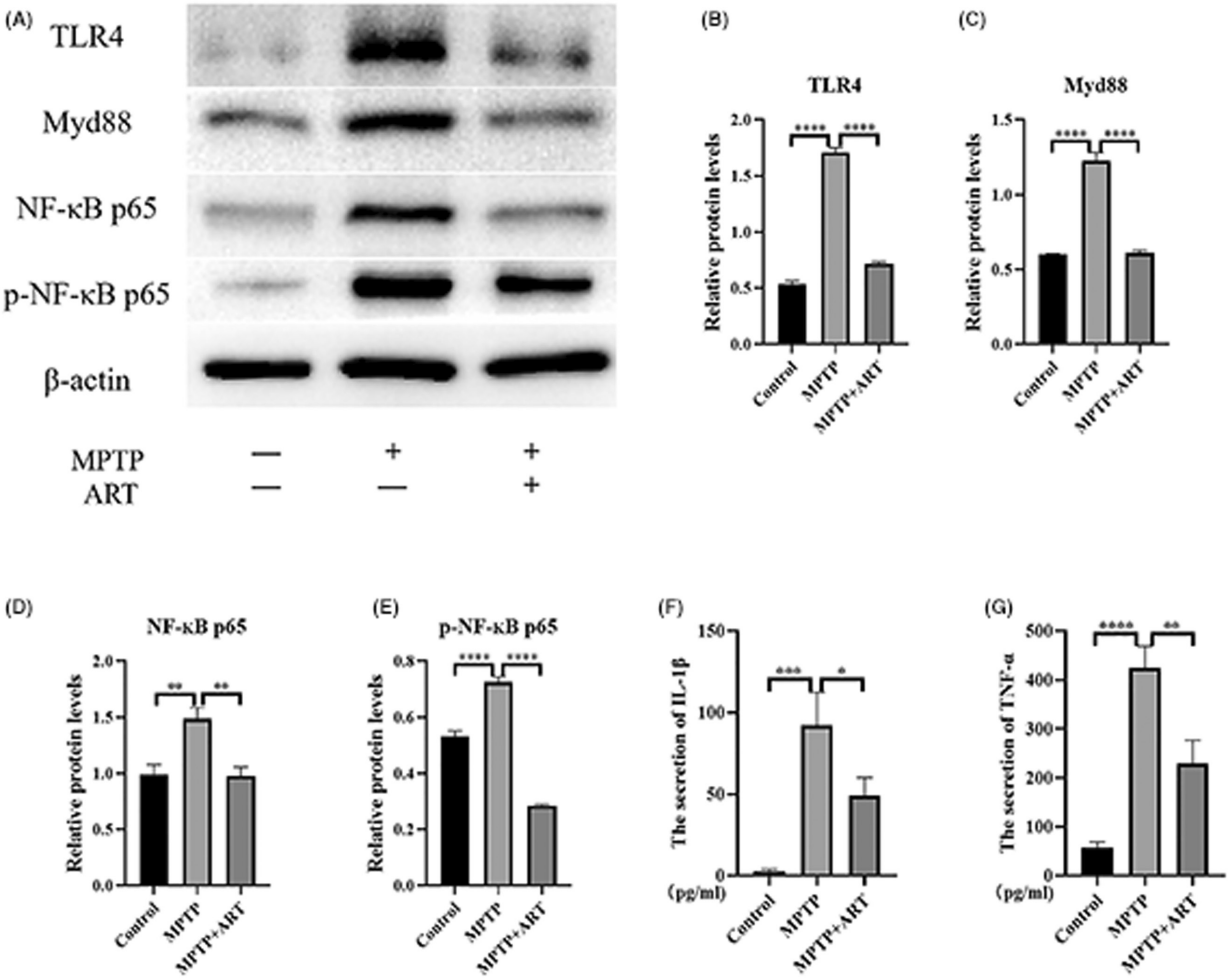
Changes in the TLR4/Myd88/NF‐ĸB pathway in artemisinin‐treated PD model mice. (A) WB results showing the expression of TLR4/Myd88/NF‐ĸB pathway components in the mouse model. (B) WB results showing that the expression of TLR4 was increased in the MPTP group compared with the blank group (0.5341 ± 0.0297 (Control) vs. 1.709 ± 0.042 (MPTP), ****p < 0.0001) and decreased after artemisinin treatment (0.717 ± 0.02088 (Artemisinin) vs. 1.709 ± 0.042 (MPTP), ****p < 0.0001). (C) WB results showing that the expression of Myd88 was increased in the MPTP group compared with the blank group (0.6015 ± 0.003775 (Control) vs. 1.227 ± 0.0553 (MPTP), ****p < 0.0001) and decreased after artemisinin treatment (0.6123 ± 0.01769 (Artemisinin) vs. 1.227 ± 0.0553 (MPTP), ****p < 0.0001). (D) WB results showing that the expression of NF‐ĸB p65 was increased in the MPTP group compared with the blank group (0.9856 ± 0.0923 (Control) vs. 1.487 ± 0.096 (MPTP), **p = 0.0011 < 0.01) and decreased after artemisinin treatment (0.9764 ± 0.07912 (Artemisinin) vs. 1.487 ± 0.096 (MPTP), **p = 0.001 < 0.01). (E) WB results showing that the expression of p‐NF‐ĸB p65 was increased in the MPTP group compared with the blank group (0.53 ± 0.02066 (Control) vs. 0.7249 ± 0.0186 (MPTP), ****p < 0.0001) and decreased after artemisinin treatment (0.2853 ± 0.00419 (Artemisinin) vs. 0.7249 ± 0.0186 (MPTP), ****p < 0.0001). (F) ELISA was used to measure the content of IL‐1β in the cell supernatant in the 3 groups, and the results indicated that IL‐1β levels increased (2.407 ± 1.697 (Artemisinin) vs. 92.41 ± 20.01 (MPTP), ***p = 0.0004 < 0.001) after MPTP treatment and decreased after artemisinin treatment (49.07 ± 11.13 (Artemisinin) vs. 92.41 ± 20.01 (MPTP), *p = 0.0167 < 0.05). (G) ELISA was used to measure the content of TNF‐α in the cell supernatant in the 3 groups, and the results indicated that TNF‐α levels increased (57.22 ± 11.76 (Artemisinin) vs. 424.6 ± 44.53 (MPTP), ****p < 0.0001) after MPTP treatment and decreased after artemisinin treatment (229.4 ± 47.51 (Artemisinin) vs. 424.6 ± 44.53 (MPTP), **p = 0.0019 < 0.01). ****p < 0.0001). *n* = 3, ***p < 0.001, **p < 0.01, *p < 0.05.

On the basis of the results presented in Section 3.2, the expression of TLR4/Myd88/NF‐KB‐related proteins in the midbrains of mice in the blank group, model group, and artemisinin‐treating group was analyzed by WB (Figure 6A–E). The expression levels of the TLR4/Myd88/NF‐KB‐related proteins were higher than those in the blank group and decreased after artemisinin treatment. Measurement of TNF‐α and IL‐β levels in midbrain tissues by ELISA (Figure 6F,G) showed that the expression of inflammatory factors in the model group was higher than that in the blank group and decreased after artemisinin treatment.

## 4 DISCUSSION

As the lifespan and age of the population are increasing, the number of patients with PD, which is one of the most common diseases in the elderly population, is also increasing year by year. As our understanding of PD pathogenesis has improved, we have identified the involvement of many mechanisms, including oxidative stress,^16^ mitochondrial dysfunction,^17^ the inflammatory response,^18^ and excitatory amino acid toxicity.^19^ The ultimate consequence of these mechanisms is DAergic neuron injury in the SNpc. There is not enough DOPA in the CNS to dynamically balance acetylcholine after DAergic neuron injury in the SNpc. PD manifests as a series of clinical extrapyramidal symptoms that seriously affect the quality of life of patients and their families. DAergic neuron damage is related to the CNS microenvironment and associated factors, and microglial cells play a key role in this process.^20^

Microglial cells, the innate immune cells of the CNS, monitor the microenvironment of the CNS. They play an important role in maintaining normal brain function. When the brain microenvironment changes, resting microglial cells become activated and release a variety of substances, such as cytokines, chemokines, and growth factors. These released substances maintain microenvironmental homeostasis^21^, ^22^ in the CNS and damage brain neurons.^21^, ^23^, ^24^ As the body ages, CNS function and the ability to maintain homeostasis are impaired, and microglial cell metabolism is also altered. This is one of the reasons why the incidence of PD increases with age. It has been found that the occurrence and development of many CNS diseases are related to the activation of microglial cells.^25^, ^26^ Some studies have found that the inhibition of microglial cell activity can alleviate the symptoms of PD,^27^ and interfering with the function of microglial cells also has a therapeutic effect on PD. Many mechanisms are related to microglial cell activation, the most common of which is the activation of receptors on the microglial cell membrane. TLR4 is a membrane protein related to the activation of microglial cells.^28^, ^29^

TLR4 usually exists as a homodimer on the membranes of immune cells such as macrophages and B cells. Some studies have shown that many diseases are associated with TLR4 activation.^6^, ^30^ Overexpression of TLR4, which is mainly expressed on the microglial cell membrane, can lead to CNS diseases.^7^, ^8^, ^31^ Animal experiments have shown that the activation of microglial cells is reduced in the brains of TLR4‐deficient mice,^32^, ^33^ and this was also confirmed in cell experiments.^34^, ^35^ After overexpression of TLR4, the expression of the downstream protein myd88 increases, increasing the phosphorylation of NF‐KB‐P65 and thus elevating the expression of inflammatory factors such as IL‐1β and TNF‐α. This is consistent with the finding that serum levels of inflammatory factors are elevated in PD patients.^36^ Excessive cell activation and increased expression of inflammatory factors cause neuronal cell damage.^22^, ^37^ Therefore, TLR4 may be an important target for inhibiting microglial cell activation. We should identify drugs that can alleviate DAergic injury in PD by targeting TLR4.

Natural plants contain bioactive substances that have a therapeutic effect on PD.^38^ For example, *Mucuna pruriens*, which contains ursolic acid, can reduce the behavioral abnormalities of MPTP‐induced model animals and reduce oxidative stress in the SNpc.^39^ *Withania somnifera*, which contains chlorogenic acid, can ameliorate mitochondrial abnormalities and improve apoptosis in MPTP‐induced Parkinson's disease model mice, and it also inhibits the expression of iNOS to exert antiinflammatory effects and improve the apoptosis of dopaminergic neurons in the SNpc in the paraquat‐induced Parkinson's disease model.^39^ Unlike these two substances, artemisinin is widely used in the clinic. Artemisinin derivatives have been found to compensate for the clinical disadvantages of artemisinin itself. Many derivatives of artemisinin have been developed, and artemisinin is easy to obtain and economical. A drug study found that long‐term use of artemisinin led to no obvious serious adverse reactions.^40^ In addition to antimalarial effects, artemisinin has therapeutic effects on diseases such as kidney disease^41^ and liver disease.^42^ However, the mechanism of action underlying its neuroprotective effects is controversial.^43^, ^44^, ^45^ Our animal experiments showed that the protective effect of artemisinin may be related to inhibition of microglial activation. Moreover, our study confirmed that artemisinin can inhibit microglial activation through the TLR4/Myd88/NFKB pathway. Finally, animal experiments showed that the expression of TLR4/Myd88/NFKB pathway components changed in the 3 groups and that TLR4, Myd88, and NFKB were only existed on the microglial membrane. However, these findings cannot explain the relationship between microglial activation and DAergic neuron damage, which requires further study.

## 5 CONCLUSION AND FUTURE PROSPECTIVE

At present, treatments for PD mainly aim to alleviate the progression of the disease and improve the quality of life of patients, and more comprehensive treatments are needed, as the efficacy of these treatments is limited, and there are no cures. A number of studies have shown that microglial activation plays a key role in PD. Therefore, we can ameliorate DAergic effects in PD by inhibiting microglial activation. Artemisinin is one of many Chinese herbal medicines with wide clinical applications and few side effects. We have demonstrated that artemisinin is effective in reducing midbrain DAergic neuron damage by inhibiting microglial activation in vitro and in vivo. Although further confirmation of these findings is needed, artemisinin may become a clinical drug for the treatment of PD in the future.

## AUTHOR CONTRIBUTIONS

All the authors had full access to all study data and take responsibility for the integrity of the data and the accuracy of the data analyses. Yunfu Wang, Jing Lv, and Jing Zhu contributed to study conception, design, and data analysis. Jing Lv, Jing Zhu, Peihan Wang, Tongyu Liu, Zhifeng Zhang, Huan Yin, Yiran Lan, Qiang Sun, Guoda Ding, Chenxi Zhou, Huajie Wang, and Zihan Wang contributed to material preparation, model establishment, data collection, and analysis. Yunfu Wang, Jing Lv, Jing Zhu, and Jiang Yuan contributed to discussion. The first draft of the manuscript was written by Jing Lv and Jing Zhu, and all the authors commented on previous versions of the manuscript.

## FUNDING INFORMATION

This study was supported by the National Natural Science Foundation of China (No. 81671238); the Cultivating Project for Young Scholar at Hubei University of Medicine (2020QDJZR013); the Scientific and Technological Project of Shiyan City of Hubei Province (21Y42).

## CONFLICT OF INTEREST

None.

## Supporting information


Supinfo01

Supinfo02

## Data Availability

The data that support the findings of this study are available from the corresponding author upon reasonable request.
