# Supplementary material for: Artemisinin exerts a protective effect in the MPTP mouse model of Parkinson's disease by inhibiting microglial activation via the TLR4/Myd88/NF‐KB pathway
Source: CNS Neurosci Ther. 2023 Jan 24;29(4):1012–23. doi: 10.1111/cns.14063 (PMC10018080; doi:10.1111/cns.14063)
Supplement: Supplementary file 1 — Supinfo01 [file CNS-29-1012-s002.pdf]

	Figure 1 pole test(mm/s)		
	control	MPTP	ART+MPTP
	477.73	344.53	468.87
	534.82	387.19	526.46
	664.55	306.99	532.21
	628.95	402.95	604.58
	558.14	383.69	452.47
Mean	572.8	365.1	516.9
SD	74.61	38.94	60.15
Result	Control vs. MPTP:***p=0.0004		
	MPTP vs. MPTP+ART:**p=0.0045		

Figure 1 wire hanging test(s)		
control	MPTP	ART+MPTP
6	18	11
8	25	14
6	22	12
8	27	12
9	21	13
7.4	22.6	12.4
1.342	3.507	1.14
Control vs. MPTP: ****p <0.0001		
MPTP vs. ART+MPTP: **** p<0.0001		

	Figure 2 IHC		
	control	MPTP	ART+MPTP
	198	92	134
	187	70	145
	165	67	141
Mean	188	79.25	143
SD	16.59	12.58	7.528
Result	Control vs. MPTP:****p<0.0001		
	MPTP vs. MPTP+ART:***p=0.0002		

Figure 2 WB TH		
control	MPTP	ART+MPTP
0.7376534	0.33563	0.6620973
0.74658573	0.3332	0.6593869
0.75449011	0.33584	0.6593869
0.746	0.335	0.66
0.00842	0.00147	0.00157
Control vs. MPTP: ****p <0.0001		
MPTP vs. ART+MPTP: **** p<0.0001		

	Figure 2 WB IBa-1		
	control	MPTP	ART+MPTP
	0.9413	1.672	1.2530183
	1.0612	1.853	1.3640061
	1.0728	1.898	1.3768305
Mean	1.025	1.808	1.331
SD	0.0728	0.12	0.0681
Result	Control vs. MPTP: ****p<0.0001		
	MPTP vs. ART+MPTP:**p=0.0015		

	Figure 3 ART(μM)						
	-	5	10	20	40	80	160
	100	100.943	100.4880213	92.0053239	81.9787045	74.48092	56.6814552
	100	100.878	100.1399036	91.1938442	81.3461837	73.65148	54.1193844
	100	100.667	102.7094559	90.0399791	79.3672866	71.20633	53.7458717
	100	100.912	103.1532891	90.5422881	79.3775325	71.02336	53.7632555
	100	100.443	99.2741726	89.9766698	79.0201331	70.67312	53.3828739
Mean	100	100.8	101.2	90.75	80.22	72.21	54.34
SD	0	0.212	1.69	0.8541	1.345	1.733	1.335

Result — vs. 5：ns p=0.8162； — vs. 10：ns p=.4766； — vs. 20：####p <0.0001；
— vs. 40：####p <0.0001； — vs. 80：####p <0.0001； — vs. 160：####p <0.0001；
IC50=221.92；IC5=14.28

	Figure 3 Cell Viability (% of control)		
	control	MPTP	ART+MPP+
	100	127.775	103.5696822
	100	135.159	114.5476773
	100	116.088	103.1051345
	100	118.974	109.821257
Mean	100	124.5	107.8
SD	0	8.673	5.463
Result	Control vs. MPP+:***p=0.0006；		
	MPP+ vs. MPP++ART:**p=0.0079		

Figure 3 IF IBa-1		
control	MPTP	ART+MPP+
1.728	1.475	1.567
25.895	22.549	23.79
5.198	4.44	4.716
1.59	24.08	4.785
0.1281	1.691	0.3836
Control vs. MPP+:****p<0.0001；		
MPP+ vs. MPP++ART:****p<0.0001		

Figure 3 Elisa TNFα（pg/ml）		
control	MPTP	ART+MPP+
12.034	82.175	48.36035
13.869	80.146	37.8777
14.884	74.736	35.51065

Figure 3 Elisa IL-1β（pg/ml）		
control	MPTP	ART+MPP+
6.18244	86.9976	22.11512
3.87892	82.5825	18.27592
2.83968	86.6137	21.53924

Mean	13.6	79.02	40.58
SD	1.444	3.846	6.839

Result Control vs. MPP+: ****p <0.0001
MPP+ vs. ART+MPP+: ***p=0.0001

4.3	85.4	20.64
1.711	2.446	2.07

Control vs. MPP+: ****p <0.0001
MPP+ vs. ART+MPP+: ****p<0.0001

Figure 3 cell apoptosis

	control	MPTP	ART+MPP+
	1	0.60768	1.138545953
	1	0.65504	1.132004981
	1	0.66268	1.100478469
	1	0.81818	1.063572791
Mean	1	0.6859	1.109
SD	0	0.09148	0.03434

Result Control vs. MPP+:****p<0.0001；
MPP+vs.MPP++ART:****p<0.0001

Figure 4 WB TLR4

	Control	MPP^{+}	MPP^{+}+ART	MPP^{+}+ART +LPS
	0.1888	0.9005	0.5249	1.33044778
	0.2051	0.8902	0.508	1.31125285
	0.2065	0.9067	0.5091	1.32693339
Mean	0.2001	0.8991	0.514	1.323
SD	0.0098	0.0083	0.00944	0.01022

Result Control vs. MPP:****p<0.0001；
MPP vs.MPP+ART:****p<0.0001
MPP++ART vs. MPP+ART+LPS:****p<0.0001

Figure 4 Cell Viability (% of control)

Control	MPP^{+}	MPP^{+}+ART	MPP^{+}+ART +LPS
100	117.103	109.70874	128.864078
100	112.834	114.29806	127.65508
100	114.093	109.31761	128.890392
100	117.462	109.80751	128.729548
100	118.974	109.82126	128.685374
100	116.1	110.6	128.6
0	2.54	2.083	0.516

Control vs. MPP:****p<0.0001；
MPP vs.MPP+ART:***p=0.0004
MPP++ART vs. MPP++ART+LPS：****p<0.0001

Figure 4 IF IBa-1

	Control	MPP^{+}	MPP^{+}+ART	MPP^{+}+ART +LPS
	2.45	14.82	6.686	20.195
	2.5	15.2	7.91	20.61
	2.352	14.31	6.452	19.427
Mean	2.434	14.78	7.016	20.08
SD	0.0753	0.447	0.783	0.6002

Result Control vs. MPP:****p<0.0001；
MPP vs.MPP+ART:****p<0.0001
MPP++ART vs. MPP++ART+LPS：
****p<0.0001

Figure 4 Elisa TNFα（pg/ml)

Control	MPP^{+}	MPP^{+}+ART	MPP^{+}+ART +LPS
1.69565	82.1754	48.36035	96.37765
2.2568	83.6052	49.4252	97.9608
2.0338	74.7361	35.51065	87.2476
1.995	80.17	44.43	93.86
0.2825	4.762	7.744	5.783

Control vs. MPP:****p<0.0001；
MPP vs.MPP+ART:***p=0.0002
MPP++ART vs. MPP++ART+LPS：****p<0.0001

Figure 4 Elisa IL-1β（pg/ml)

	Control	MPP^{+}	MPP^{+}+ART	MPP^{+}+ART +LPS
	5.7575	108.4	25.63608	90.2414
	5.9487	109.55	21.62214	90.62368
	5.1841	104	21.81328	85.84518
Mean	5.63	107.3	23.02	88.9
SD	0.3979	2.926	2.264	2.655

Result Control vs. MPP:****p<0.0001；
MPP vs.MPP+ART:****p<0.0001
MPP++ART vs. MPP++ART+LPS：
****p<0.0001

Figure 4 cell apoptosis

Control	MPP^{+}	MPP^{+}+ART	MPP^{+}+ART +LPS
1	0.60768	1.138546	0.93141289
1	0.65504	1.132005	0.91531756
1	0.66268	1.1004785	0.62679426
1	0.6418	1.124	0.8245
0	0.02979	0.02035	0.1714

Control vs. MPP:**p=0.0046；
MPP vs.MPP+ART:***p=0.0007
MPP++ART vs. MPP++ART+LPS：*p=0.013

Figure 5 WB TLR4

Figure 5 WB Myd88

	Control	MPP$^+$	MPP$^+$+ART	MPP$^+$+ART +LPS
	0.0748	1.148	0.3556409	0.9084373
	0.1396	1.258	0.3991598	1.0240268
	0.1323	1.284	0.3970358	1.031271
Mean	0.1155	1.23	0.3839	0.9879
SD	0.0355	0.072	0.02454	0.06892

Result Control vs. MPP:****p<0.0001；
MPP vs.MPP+ART:****p<0.0001
MPP++ART vs. MPP++ART+LPS：****p<0.0001

	Control	MPP$^+$	MPP$^+$+ART	MPP$^+$+ART +LPS
	0.9773978	2.1848	1.1484675	1.3717205
	0.9751809	2.0328	1.1397664	1.3646284
	0.976179	2.1413	1.1480532	1.3717084
Mean	0.9763	2.12	1.145	1.369
SD	0.00111	0.0783	0.004908	0.004091

Control vs. MPP:****p<0.0001；
MPP vs.MPP+ART:****p<0.0001
MPP++ART vs. MPP++ART+LPS：***p=0.0005

Figure 5 WB NF-KB

	Control	MPP$^+$	MPP$^+$+ART	MPP$^+$+ART +LPS
	0.4998	0.852	0.6693474	1.2041987
	0.5351	0.884	0.7091567	1.2404083
	0.4619	0.829	0.6506486	1.16334
Mean	0.4989	0.855	0.6764	1.203
SD	0.0366	0.028	0.02988	0.03856

Result Control vs. MPP:****p<0.0001；
MPP vs.MPP+ART:***p=0.0008
MPP++ART vs. MPP++ART+LPS：****p<0.0001

Figure 5 WB P-NF-KB

	Control	MPP$^+$	MPP$^+$+ART	MPP$^+$+ART +LPS
	0.0712667	0.3221	0.1795118	0.6430731
	0.0694791	0.3164	0.1646036	0.6051965
	0.0853549	0.3437	0.1970737	0.684502
Mean	0.07537	0.3274	0.1804	0.6443
SD	0.008696	0.0144	0.01625	0.03967

Control vs. MPP:****p<0.0001；
MPP vs.MPP+ART:***p=0.0002
MPP++ART vs. MPP++ART+LPS：****p<0.0001

Figure 5 Elisa TNFα（pg/ml）

	Control	MPP$^+$	MPP$^+$+ART	MPP$^+$+ART +LPS
	14.562	98.64	40.5384	80.529
	15.587	94.2	40.8802	78.4782
	2.2568	81.55	38.8294	81.8962
Mean	10.8	91.47	40.08	80.3
SD	7.418	8.867	1.099	1.72

Result Control vs. MPP:****p<0.0001；
MPP vs.MPP+ART:****p<0.0001
MPP++ART vs. MPP++ART+LPS：***p=0.0001

Figure 5 Elisa IL-1β（pg/ml）

	Control	MPP$^+$	MPP$^+$+ART	MPP$^+$+ART +LPS
	9.77146	112.22	25.06266	89.85912
	7.47778	111.84	24.68038	99.60726
	3.87892	108.78	24.10696	103.23892
Mean	7.043	110.9	24.62	97.57
SD	2.97	1.886	0.481	6.919

Control vs. MPP:****p<0.0001；
MPP vs.MPP+ART:****p<0.0001
MPP++ART vs. MPP++ART+LPS：****p<0.0001

Figure 6 WB TLR4

	Control	MPTP	MPTP+ART
	0.5665	1.751	0.7408797
	0.5081	1.667	0.7020216
	0.5278	1.708	0.7082116
Mean	0.5341	1.709	0.717
SD	0.0297	0.042	0.02088

Result Control vs. MPTP:****p<0.0001
MPTP vs. ART+MPTP:****p<0.0001

Figure 6 WB Myd88

	Control	MPTP	MPTP+ART
	0.6058637	1.2749	0.6320109
	0.5988663	1.2396	0.6072444
	0.5999093	1.1665	0.5977479
	0.6015	1.227	0.6123
	0.003775	0.0553	0.01769

Control vs. MPTP:****p<0.0001
MPTP vs. ART+MPTP:****p<0.0001

Figure 6 WB NF-KB

	Control	MPTP	MPTP+ART
	0.9533	1.482	0.9717039
	1.0897	1.586	1.057732
	0.9139	1.394	0.8997046
Mean	0.9856	1.487	0.9764
SD	0.0923	0.096	0.07912

Result Control vs. MPTP:**p=0.0011
MPTP vs. ART+MPTP:**p=0.001

Figure 6 WB P-NF-KB

	Control	MPTP	MPTP+ART
	0.5522722	0.738	0.2884459
	0.5114563	0.7036	0.286833
	0.5263423	0.7332	0.2805186
	0.53	0.7249	0.2853
	0.02066	0.0186	0.00419

Control vs. MPTP:****p<0.0001
MPTP vs. ART+MPTP:****p<0.0001

Figure 6 Elisa TNFα（pg/ml）

Control	MPTP	MPTP+ART

Figure 6 Elisa IL-1β（pg/ml）

Control	MPTP	MPTP+ART

	70.556	473.9	191.66667
	52.778	412.8	282.77778
	48.333	387.2	213.88889
Mean	57.22	424.6	229.4
SD	11.76	44.53	47.51
Result	Control vs. MPTP: ****p<0.0001<br>MPTP vs. ART+MPTP: **p=0.0019		

0.5555556	106.11	61.666667
2.7777778	101.67	45
3.8888889	69.444	40.555556
2.407	92.41	49.07
1.697	20.01	11.13
Control vs. MPTP: ***p=0.0004<br>MPTP vs. ART+MPTP: * p=0.0167		
