# Supplementary material for: Artemisinin exerts a protective effect in the MPTP mouse model of Parkinson's disease by inhibiting microglial activation via the TLR4/Myd88/NF‐KB pathway
Source: CNS Neurosci Ther. 2023 Jan 24;29(4):1012–23. doi: 10.1111/cns.14063 (PMC10018080; doi:10.1111/cns.14063)
Supplement: Supplementary file 2 — Supinfo02 [file CNS-29-1012-s001.pdf]

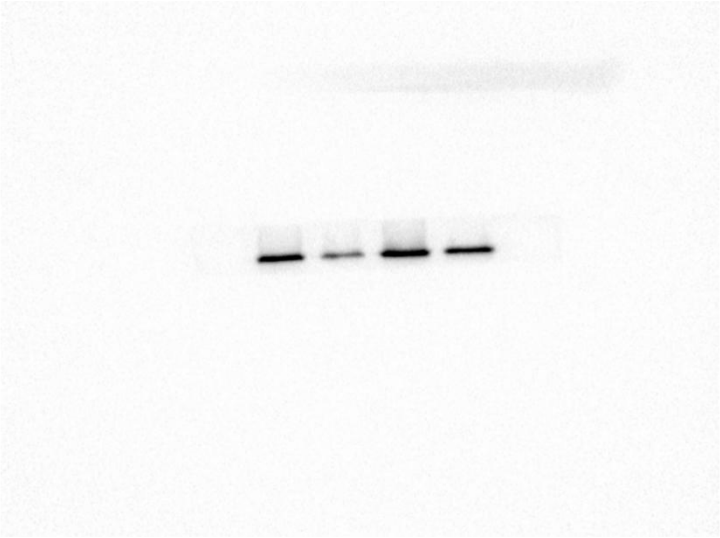

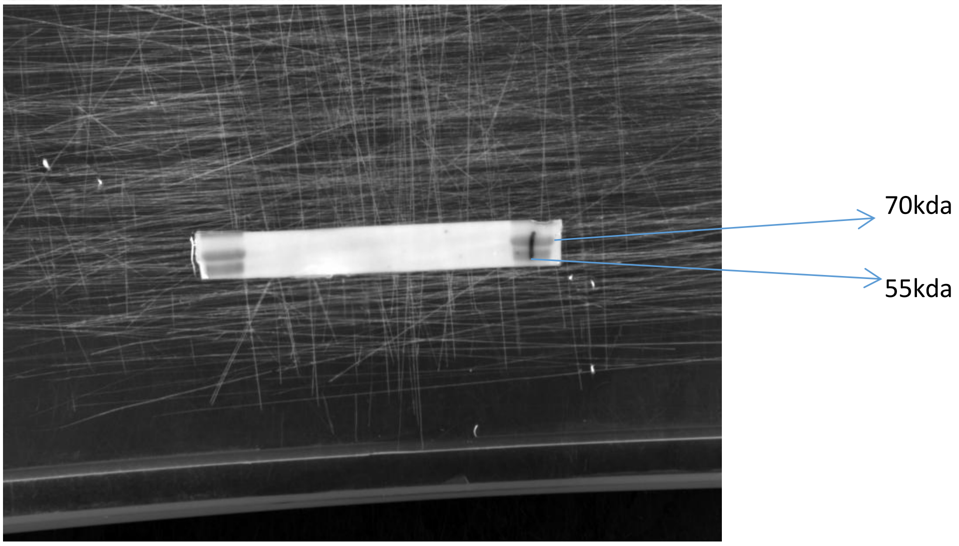

Figure2：TH(55-60KDa)

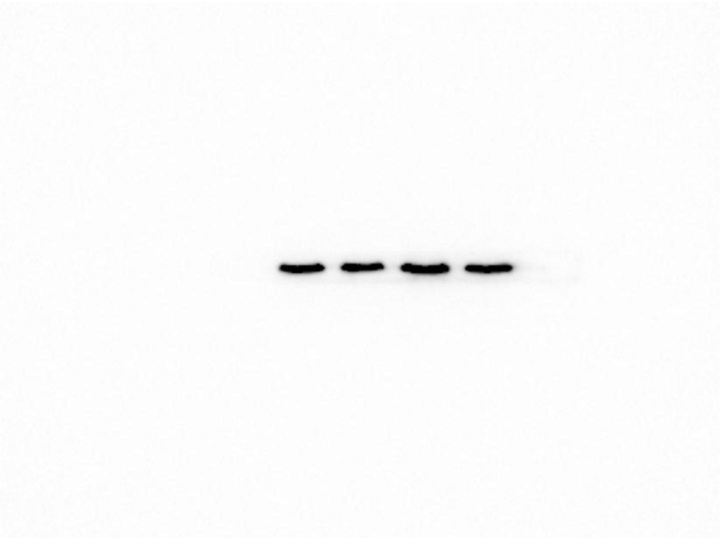

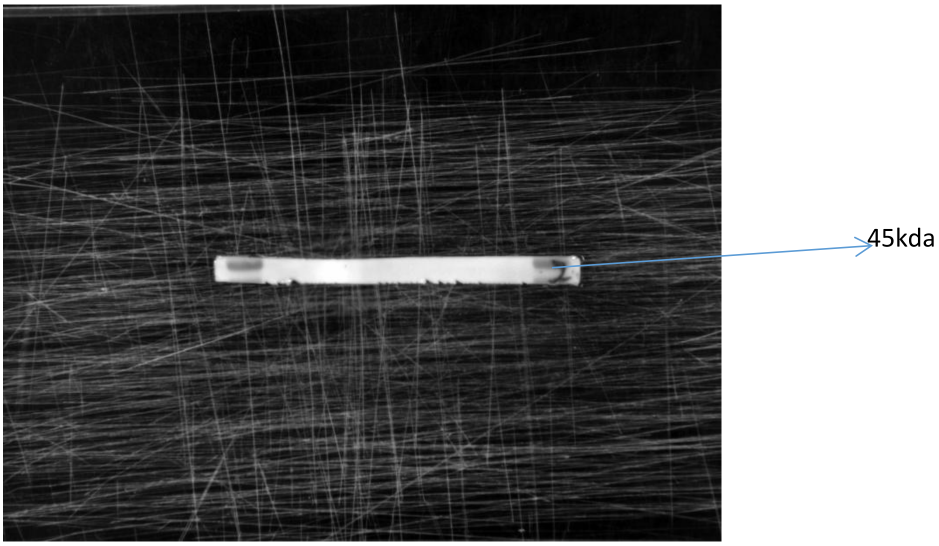

Figure2：TH-actin(42.5KDa)

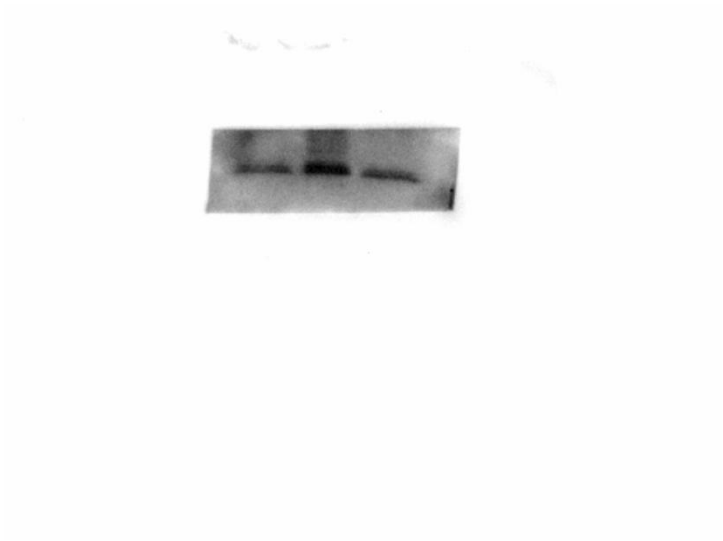

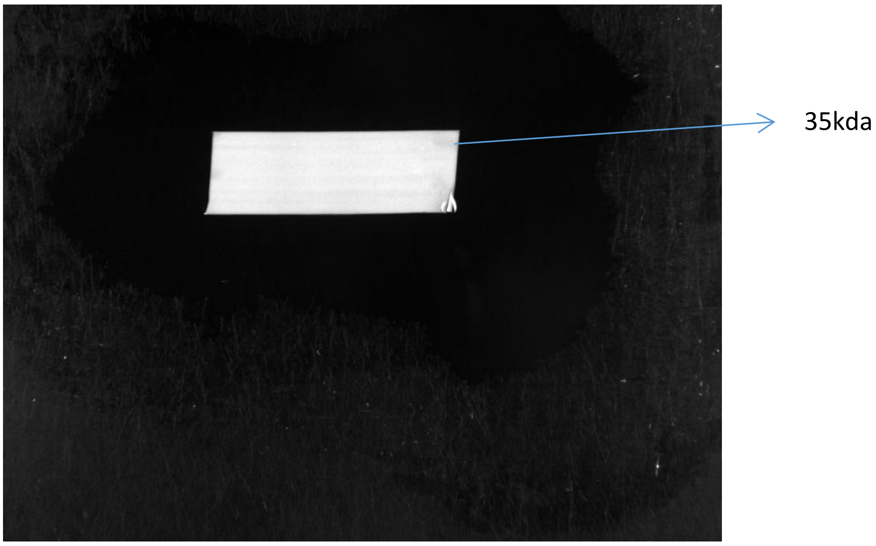

Figure2：IBa-1(17KDa)

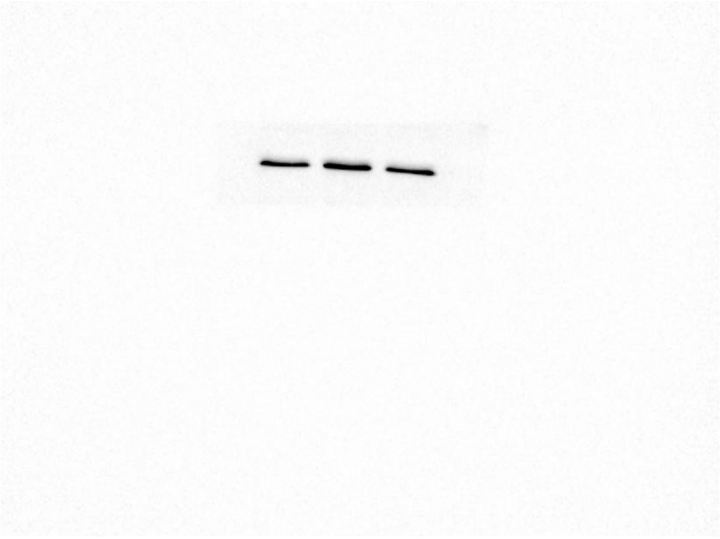

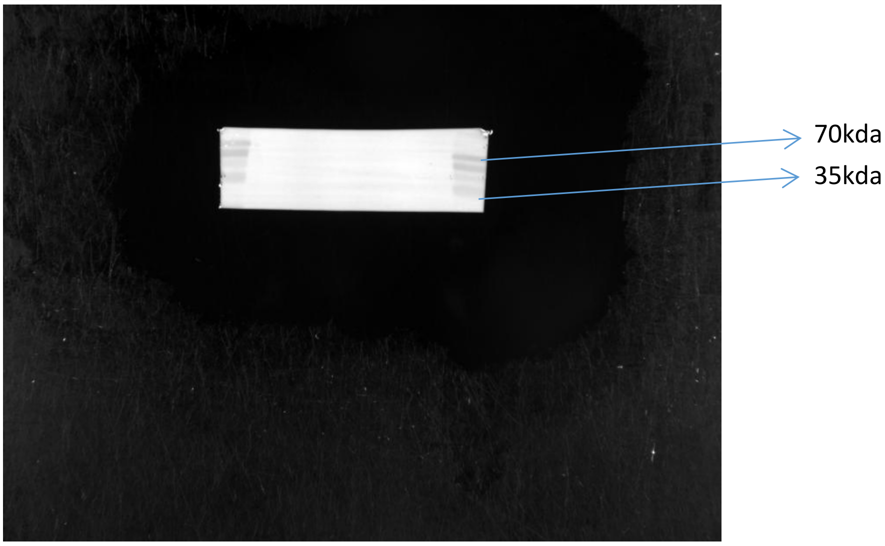

Figure2：IBa-1-actin(42.5KDa)

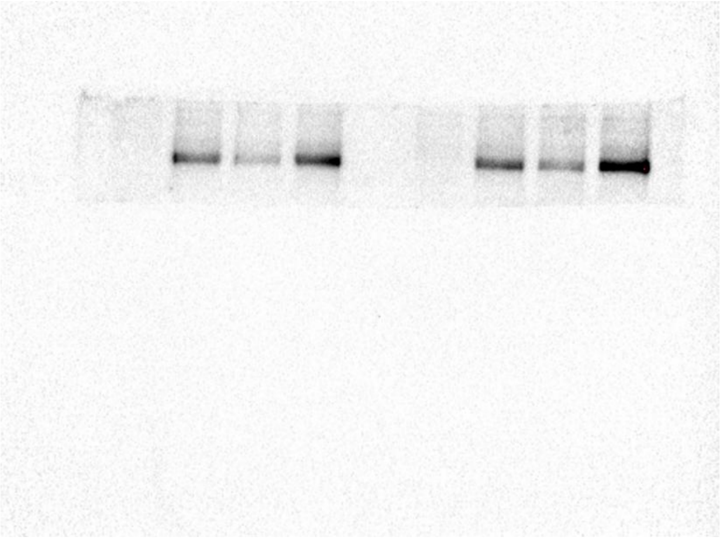

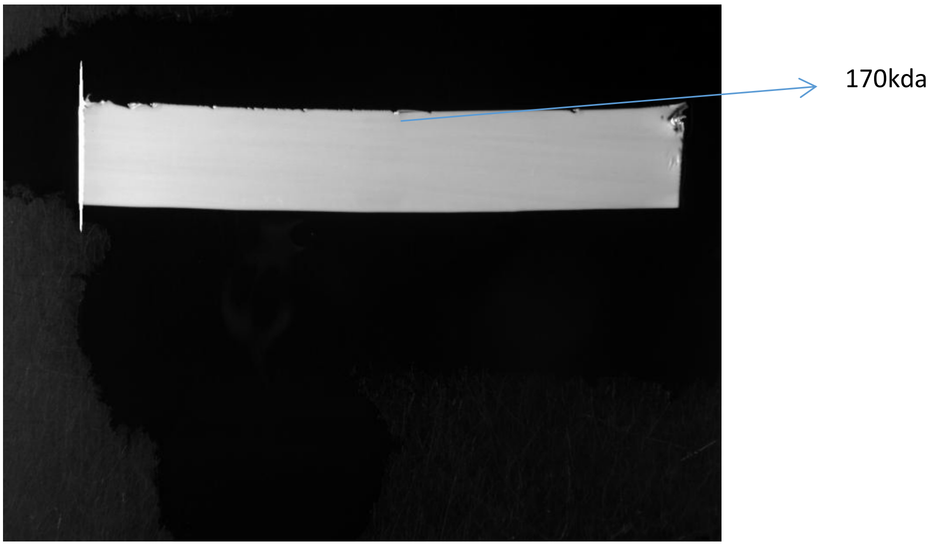

Figure4：TLR4(100-135KDa)

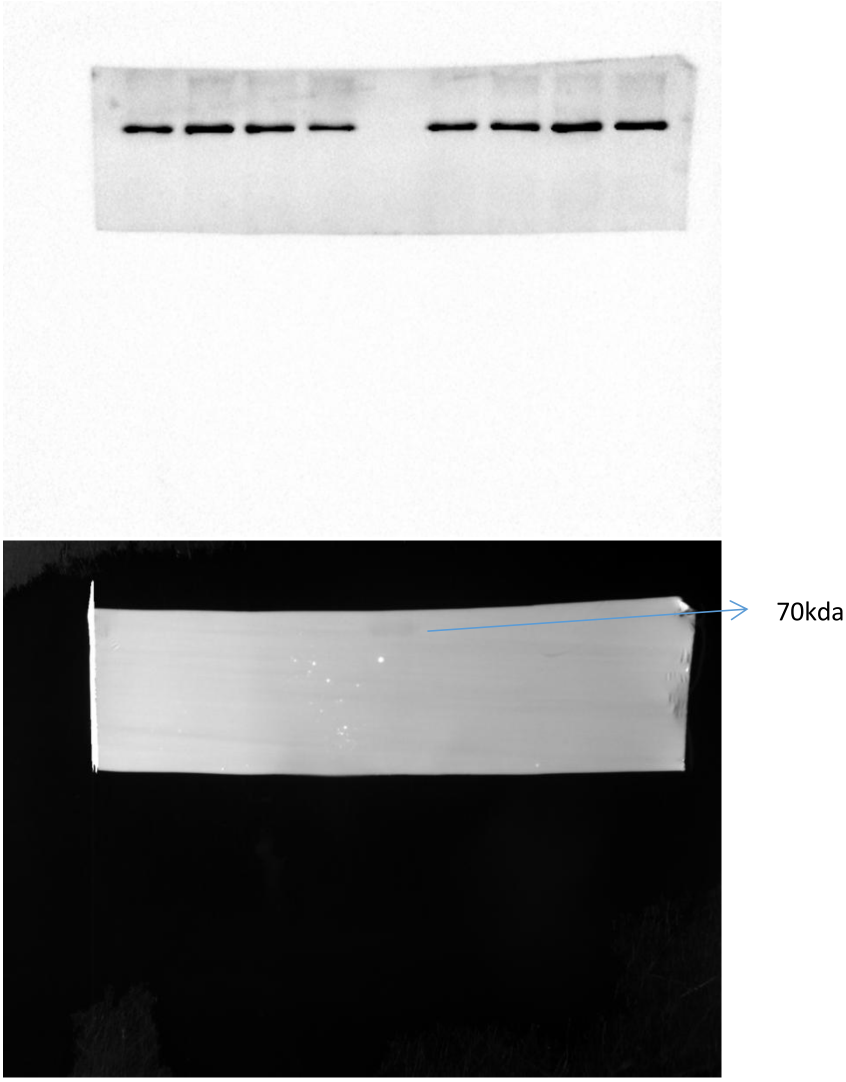

Figure4：TLR4-actin(42.5KDa)

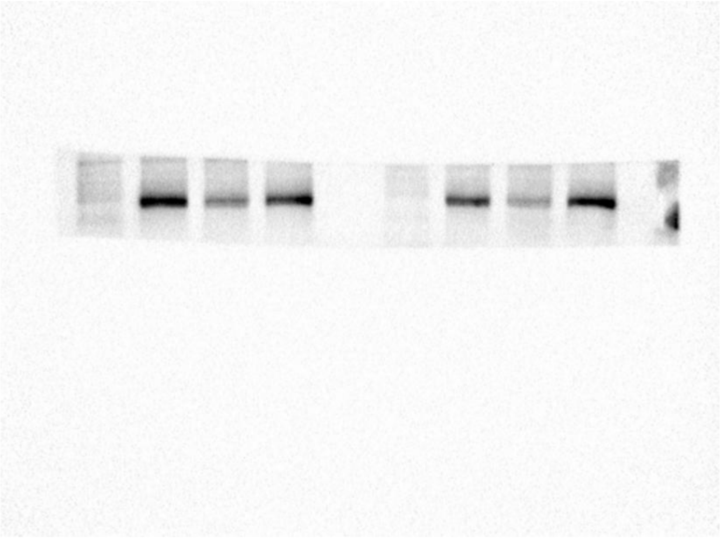

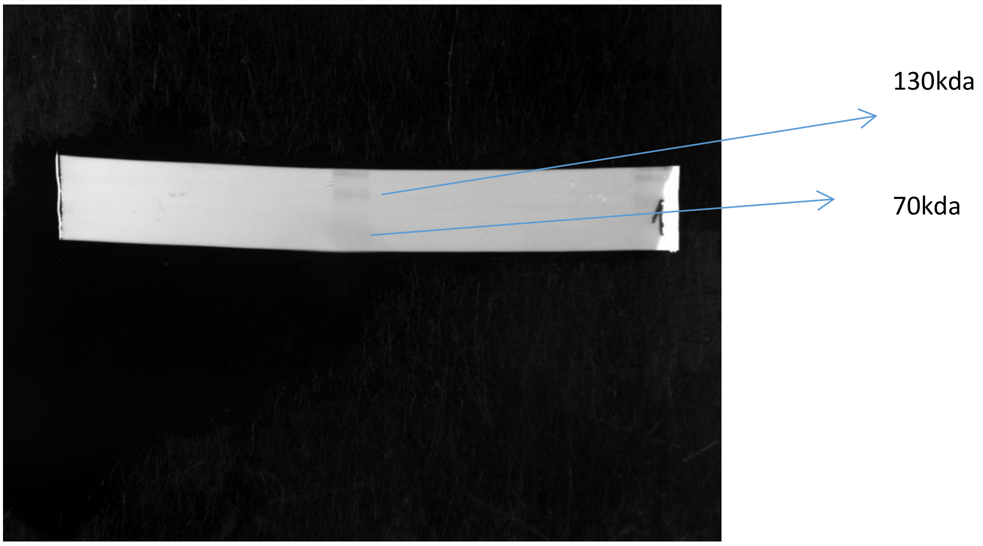

Figure5：tlr4(100-135KDa)

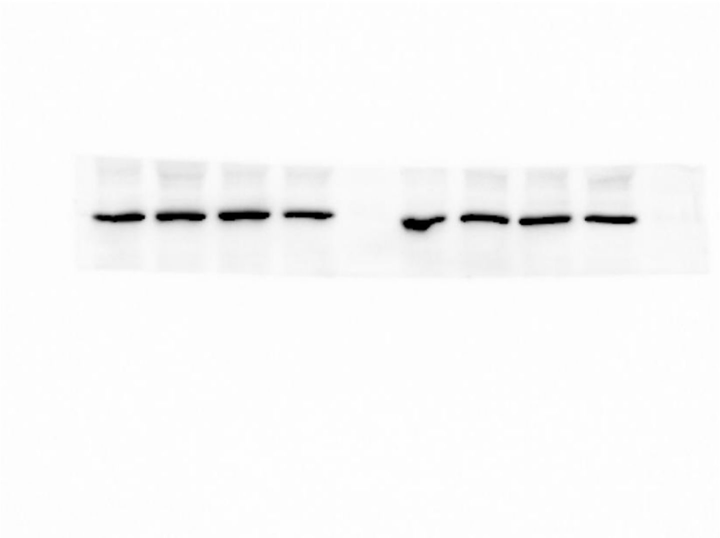

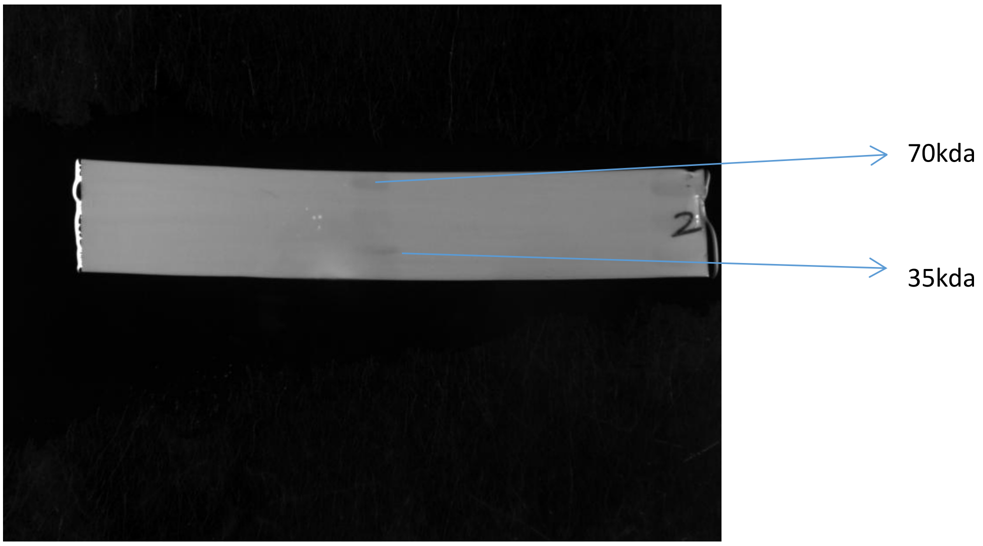

Figure5： TLR4-actin(42.5KDa)

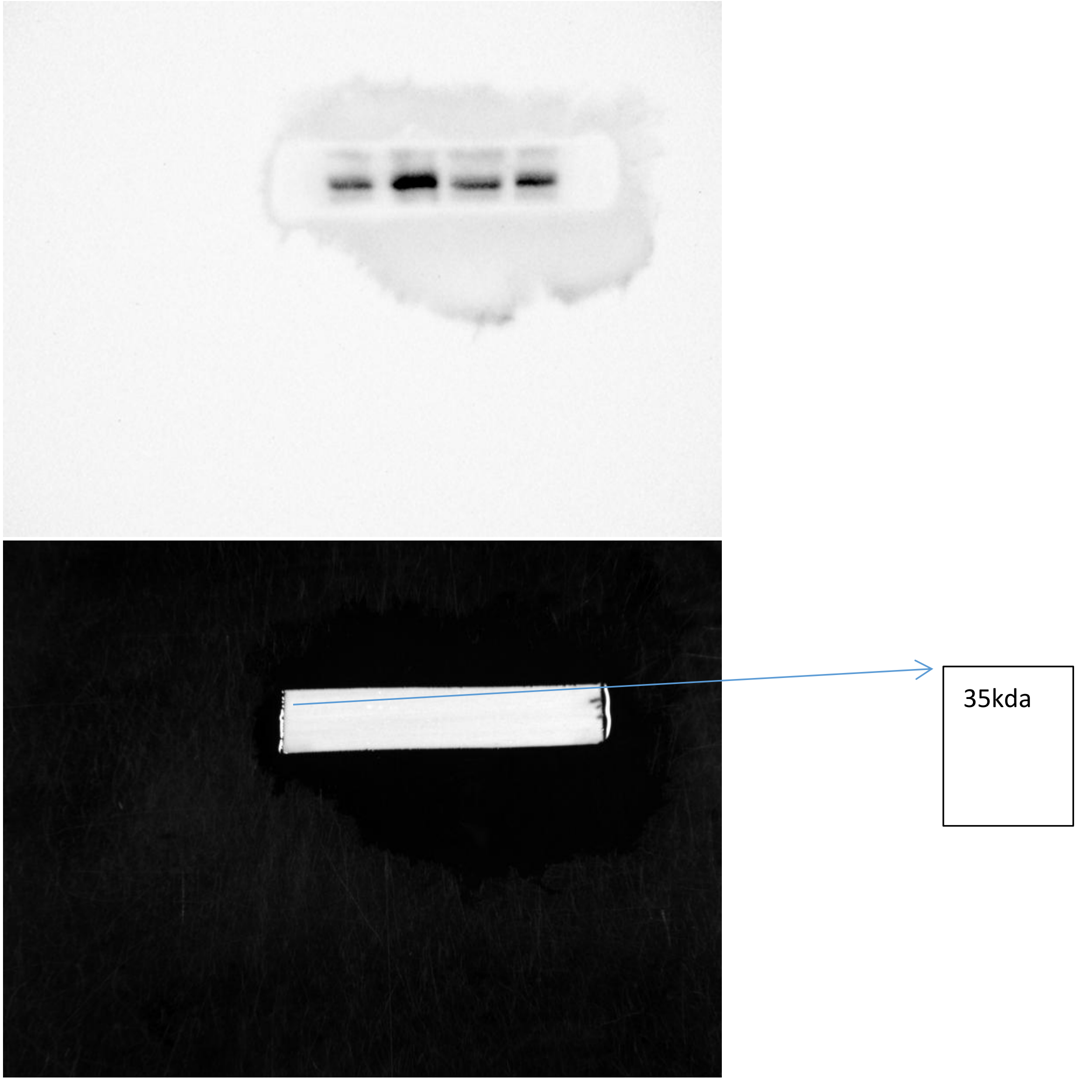

Figure5： myd88(33KDa)

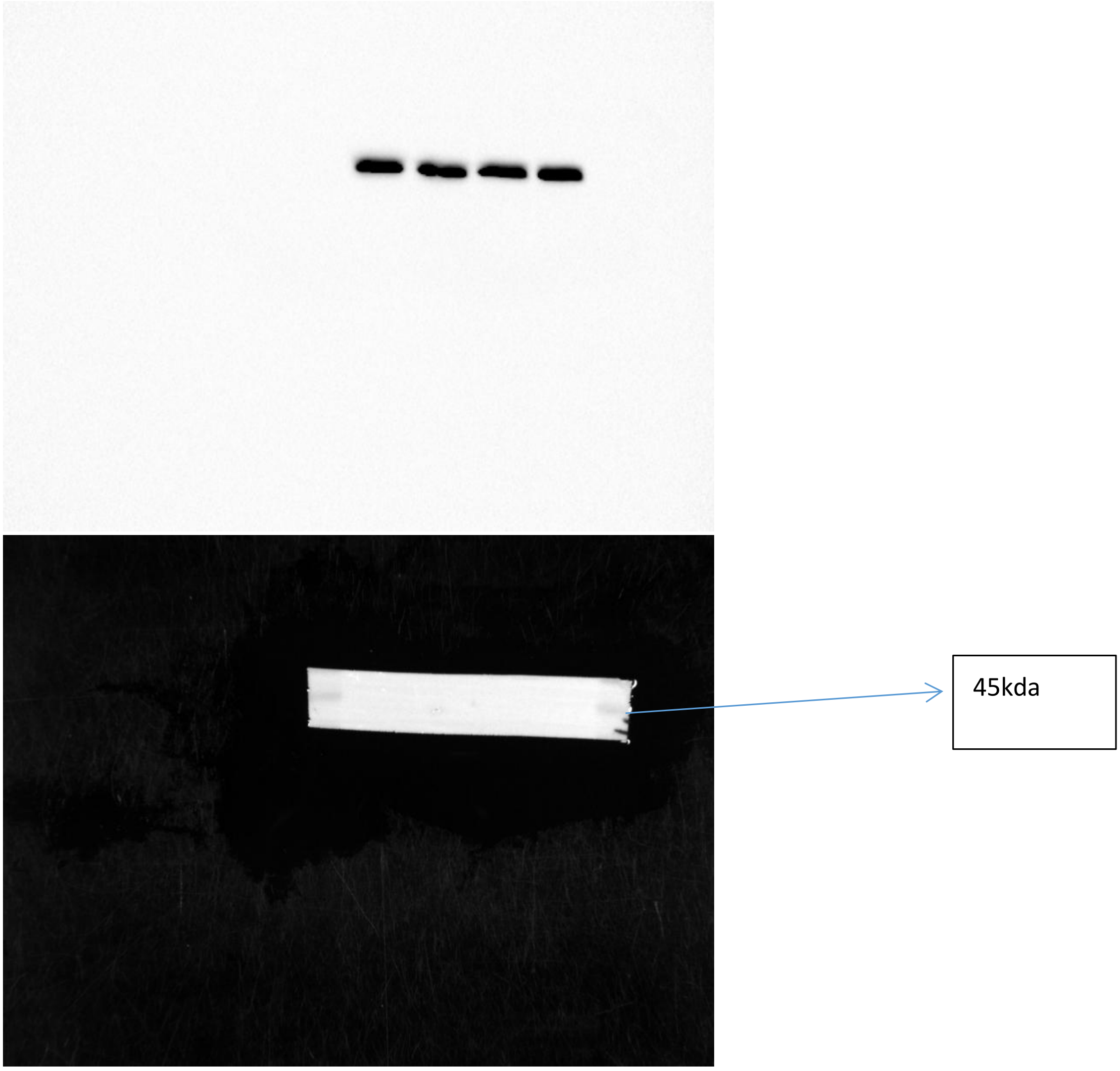

Figure5： myd88-actin(42.5KDa)

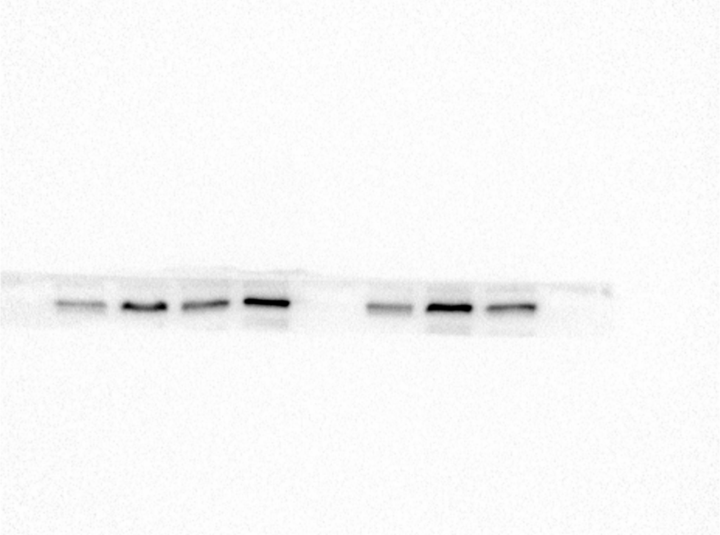

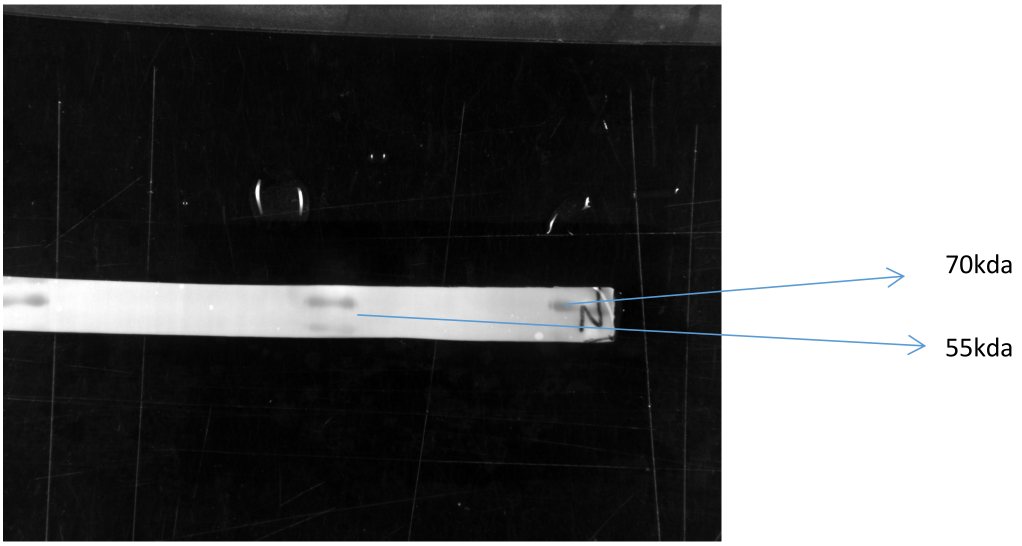

Figure5：NF-kBp65(65KDa)

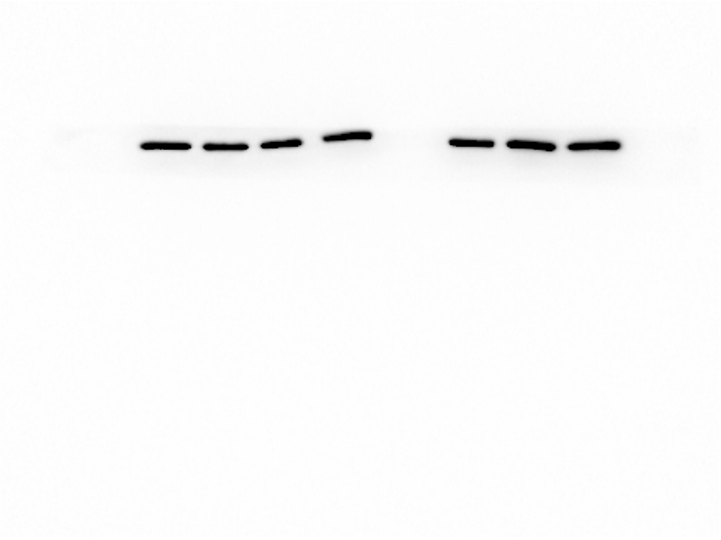

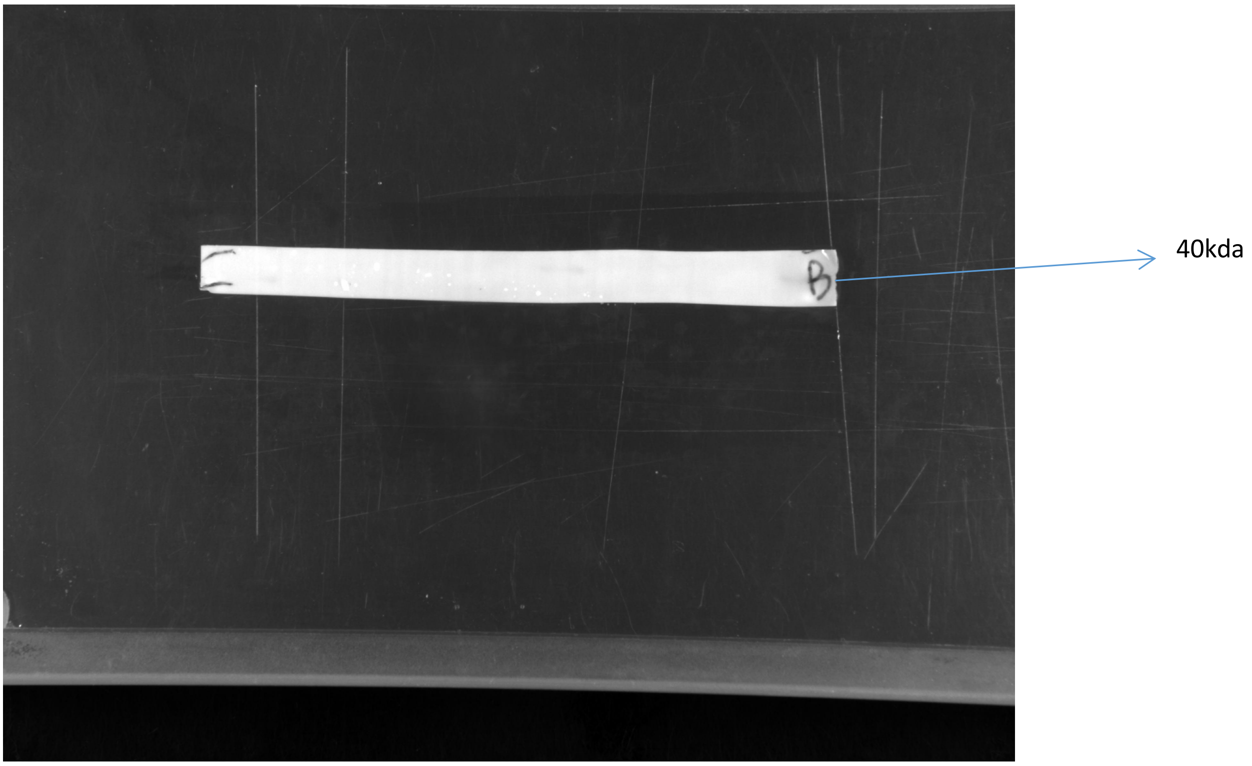

Figure5：NF-KBp65-actin(42.5KDa)

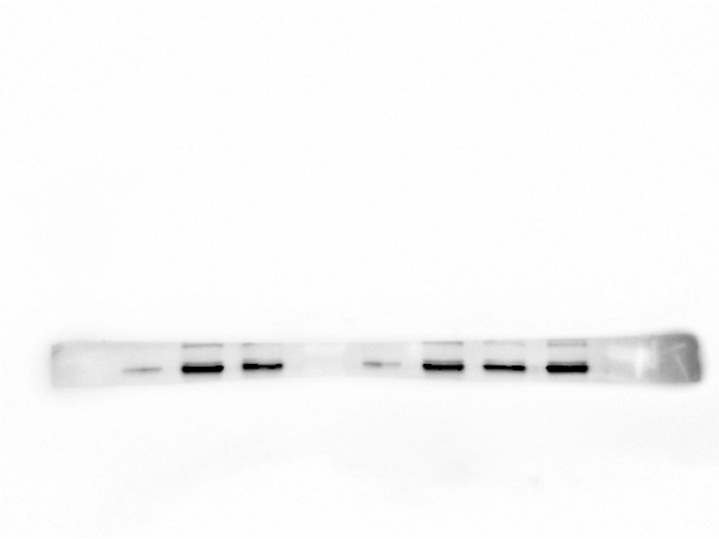

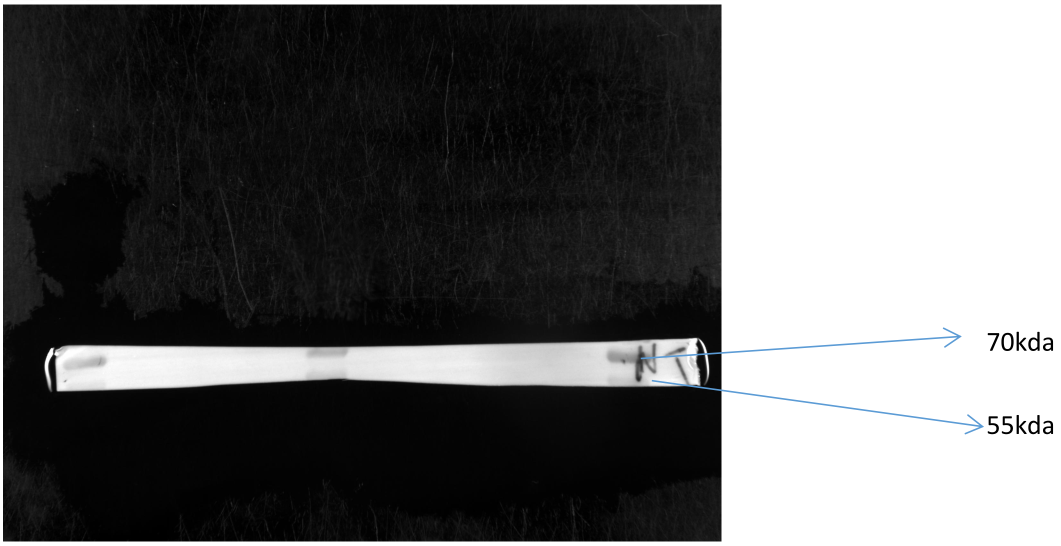

Figure5：p-NF-kB p65(65KDa)

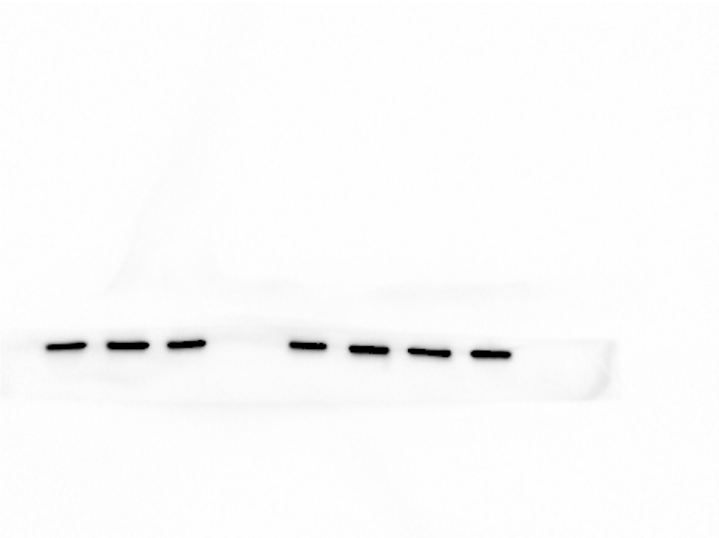

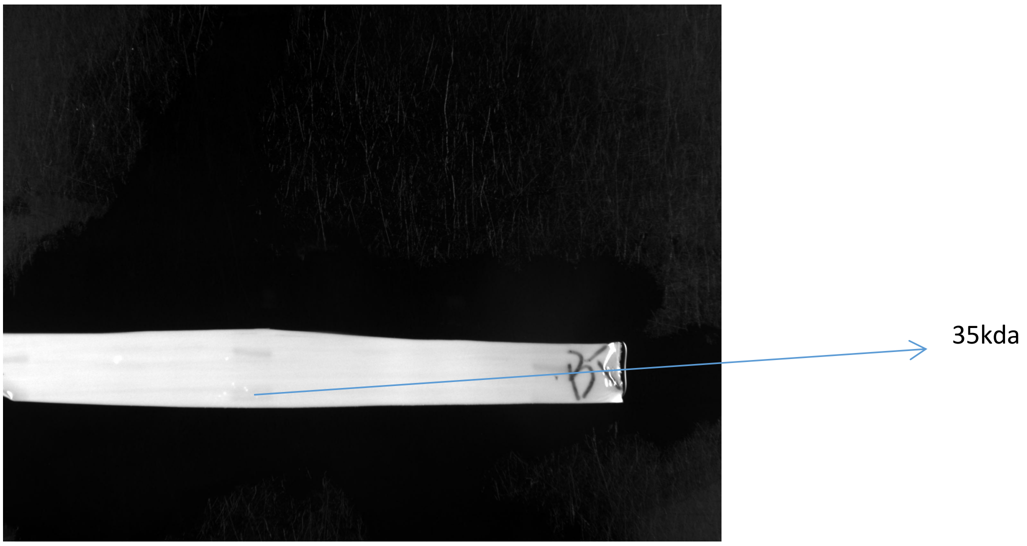

Figure5：p-NF-KB p65-actin(42.5KDa)

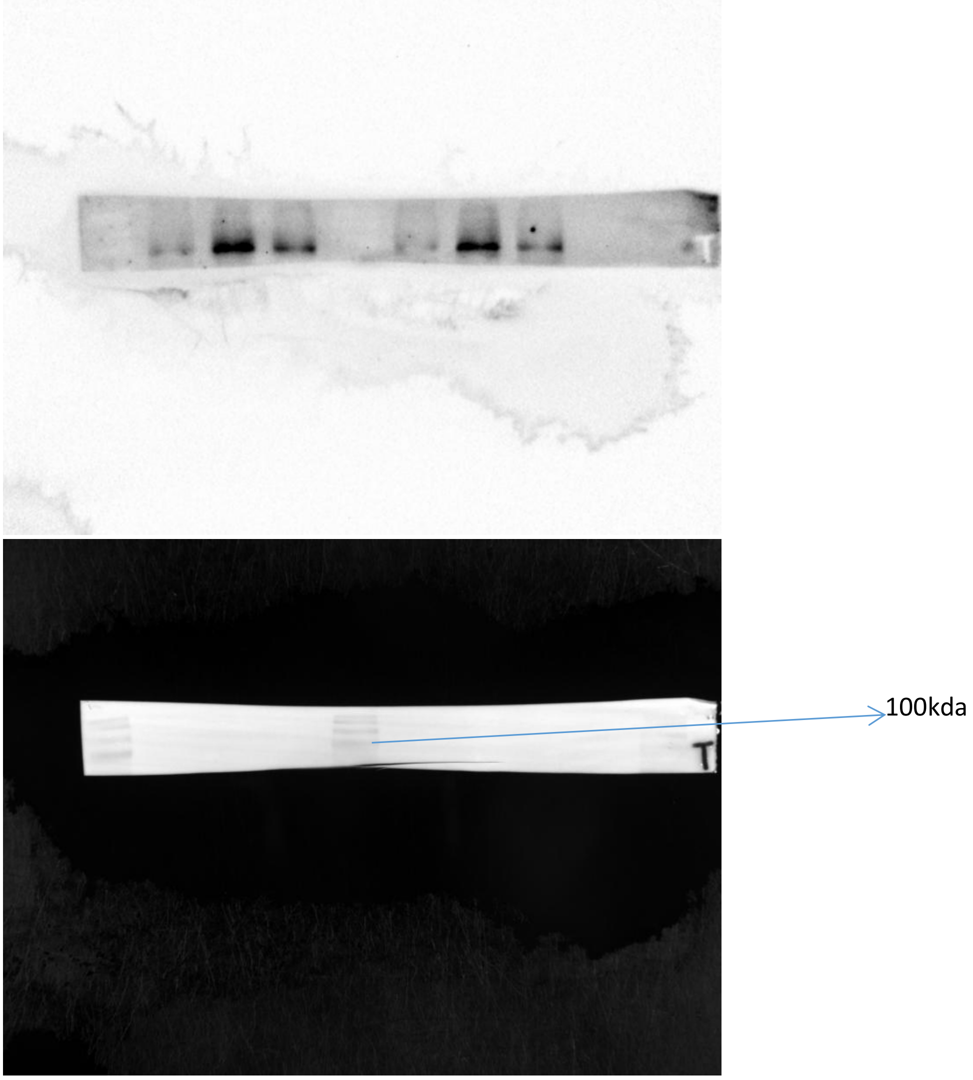

Figure6：tlr4(100-135KDa)

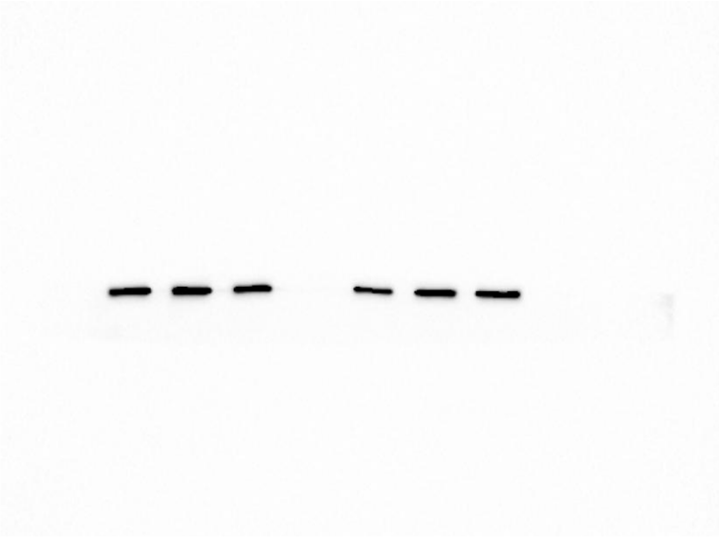

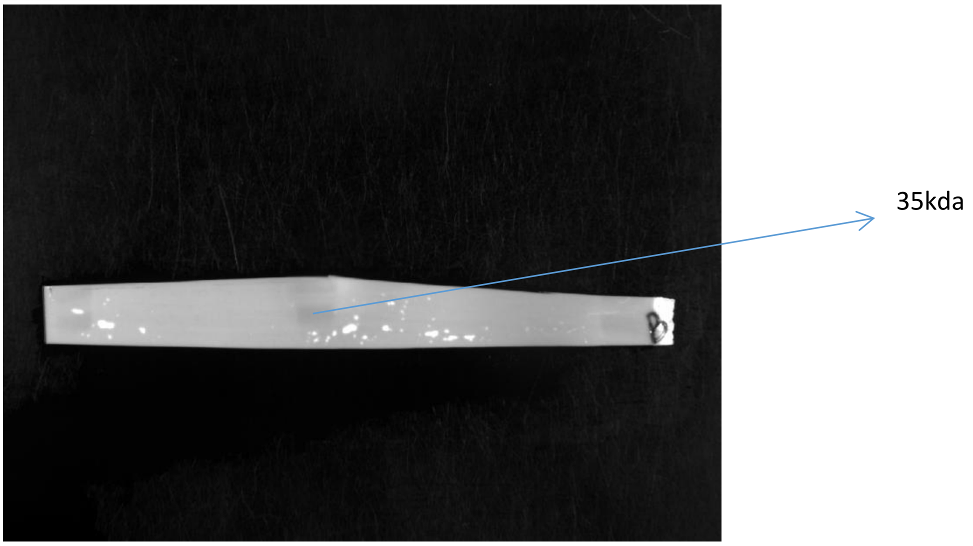

Figure6：TLR4-actin(42.5KDa)

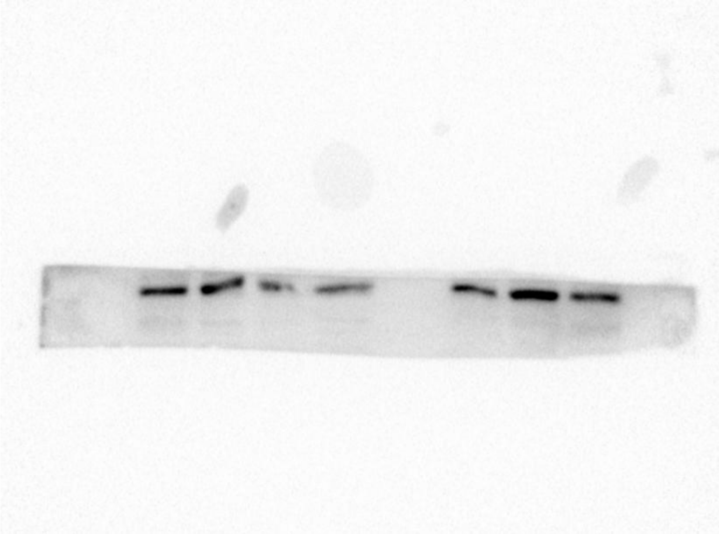

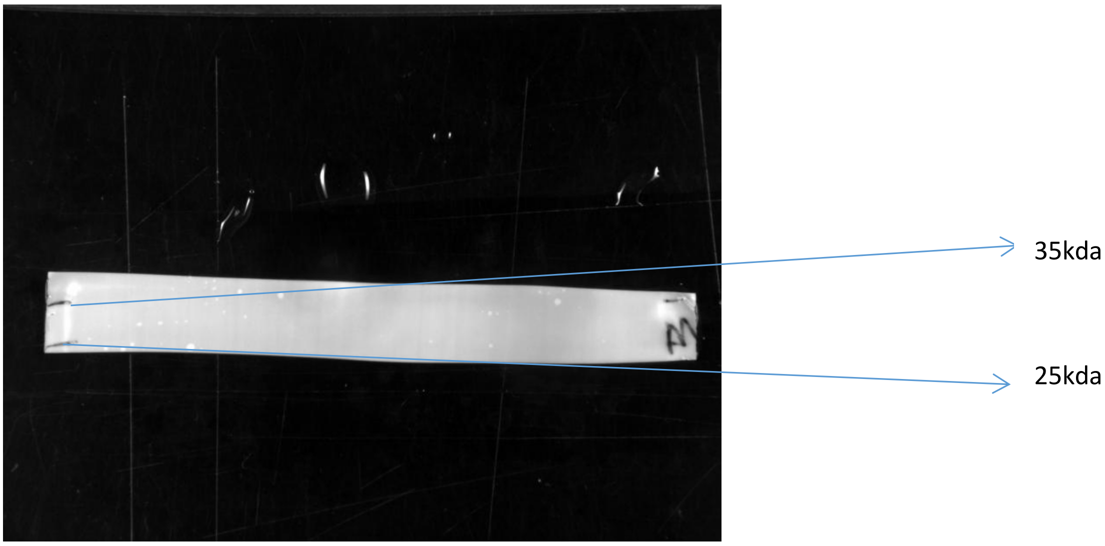

Figure6： myd88(33KDa)

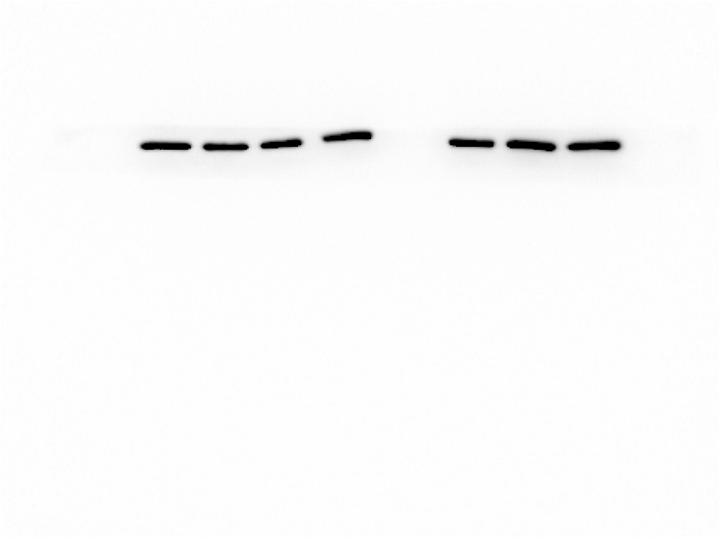

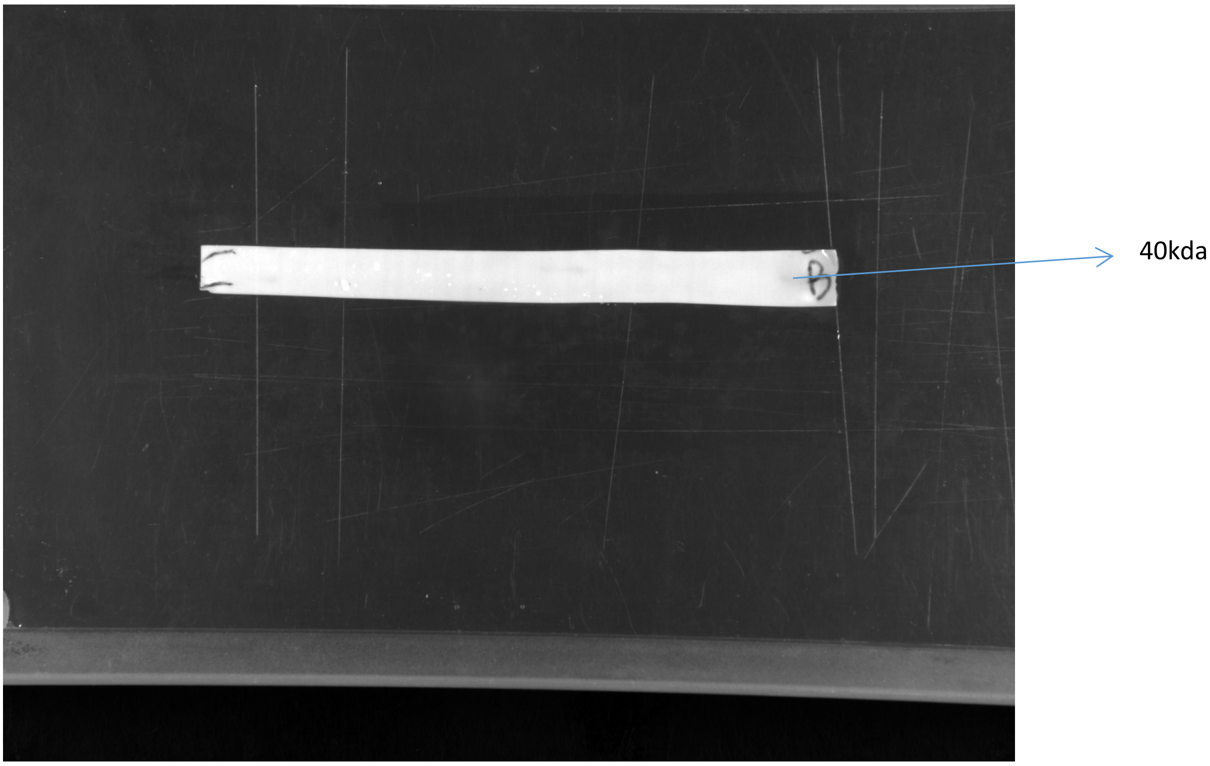

Figure6： myd88-actin(42.5KDa)

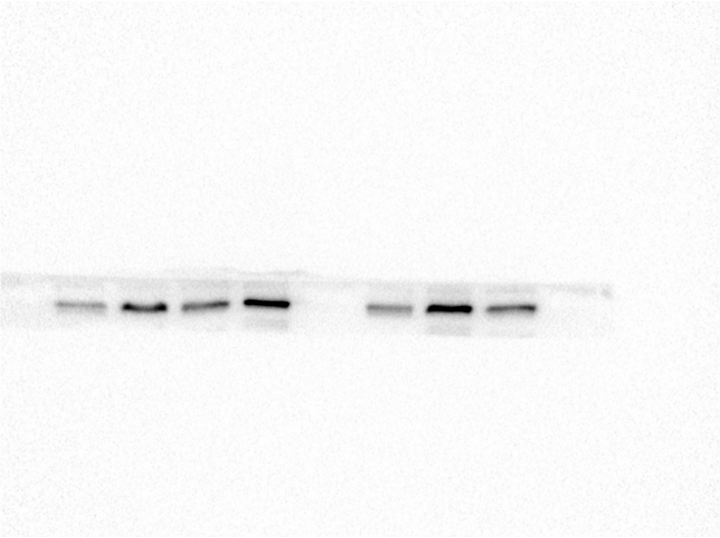

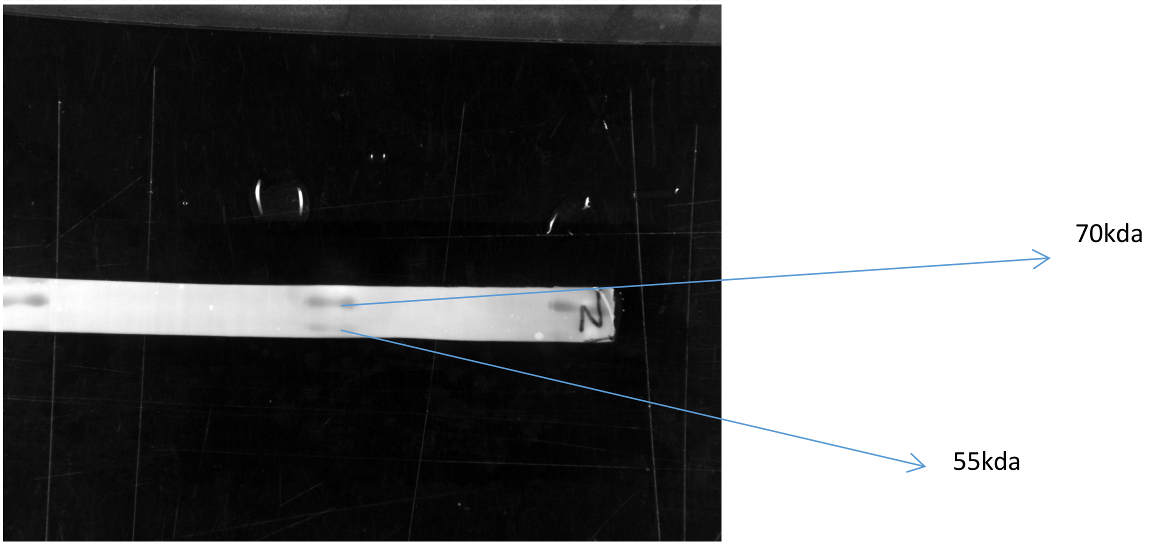

Figure6：NF-kBp65(65KDa)

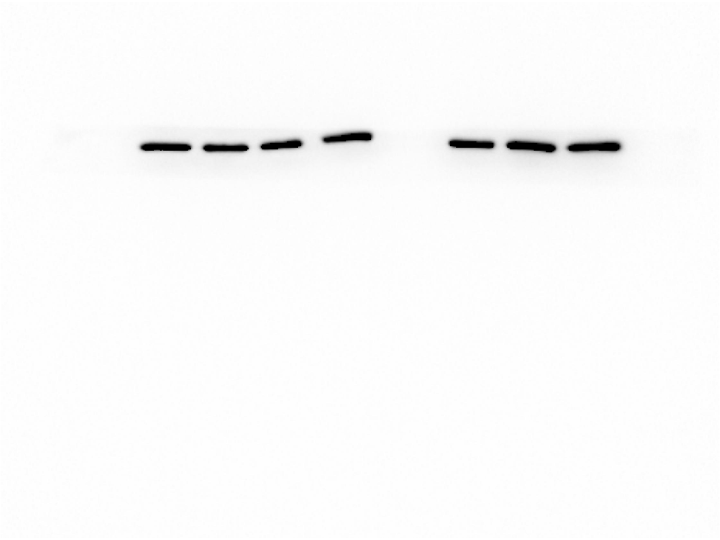

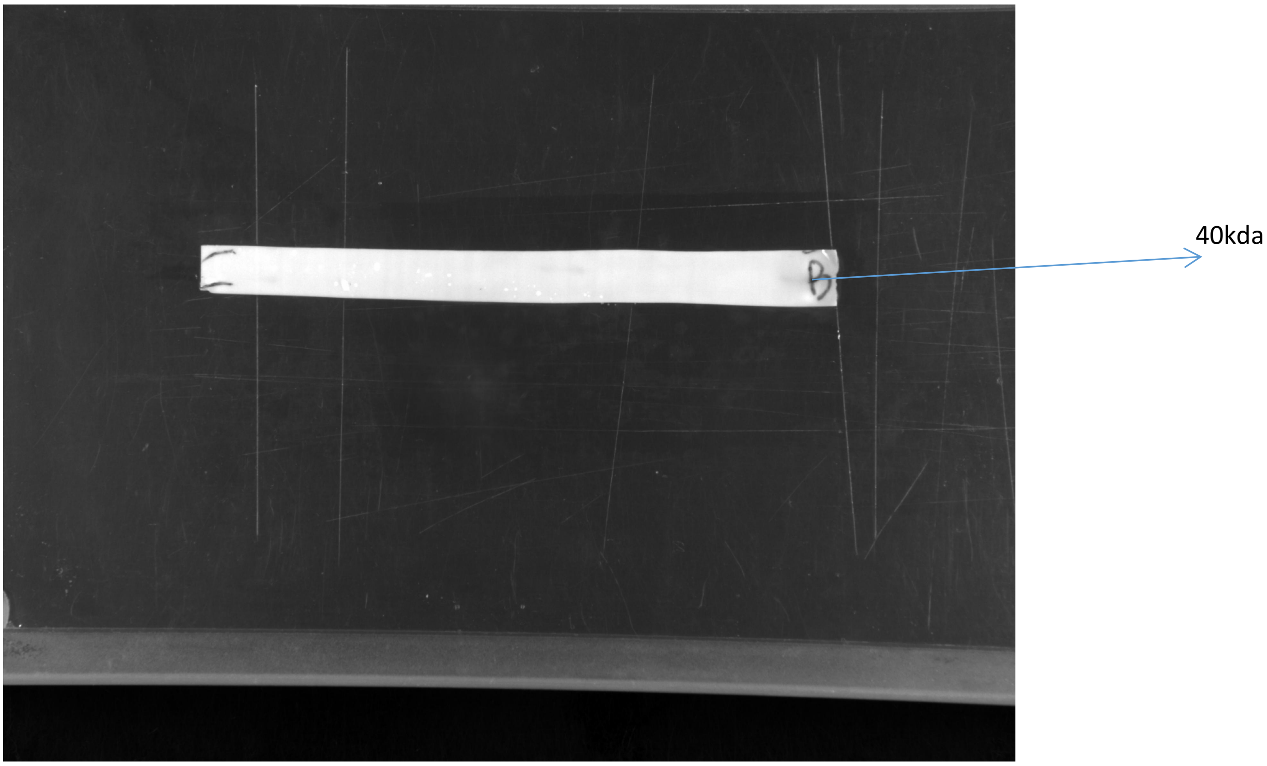

Figure6：NF-KBp65-actin(42.5KDa)

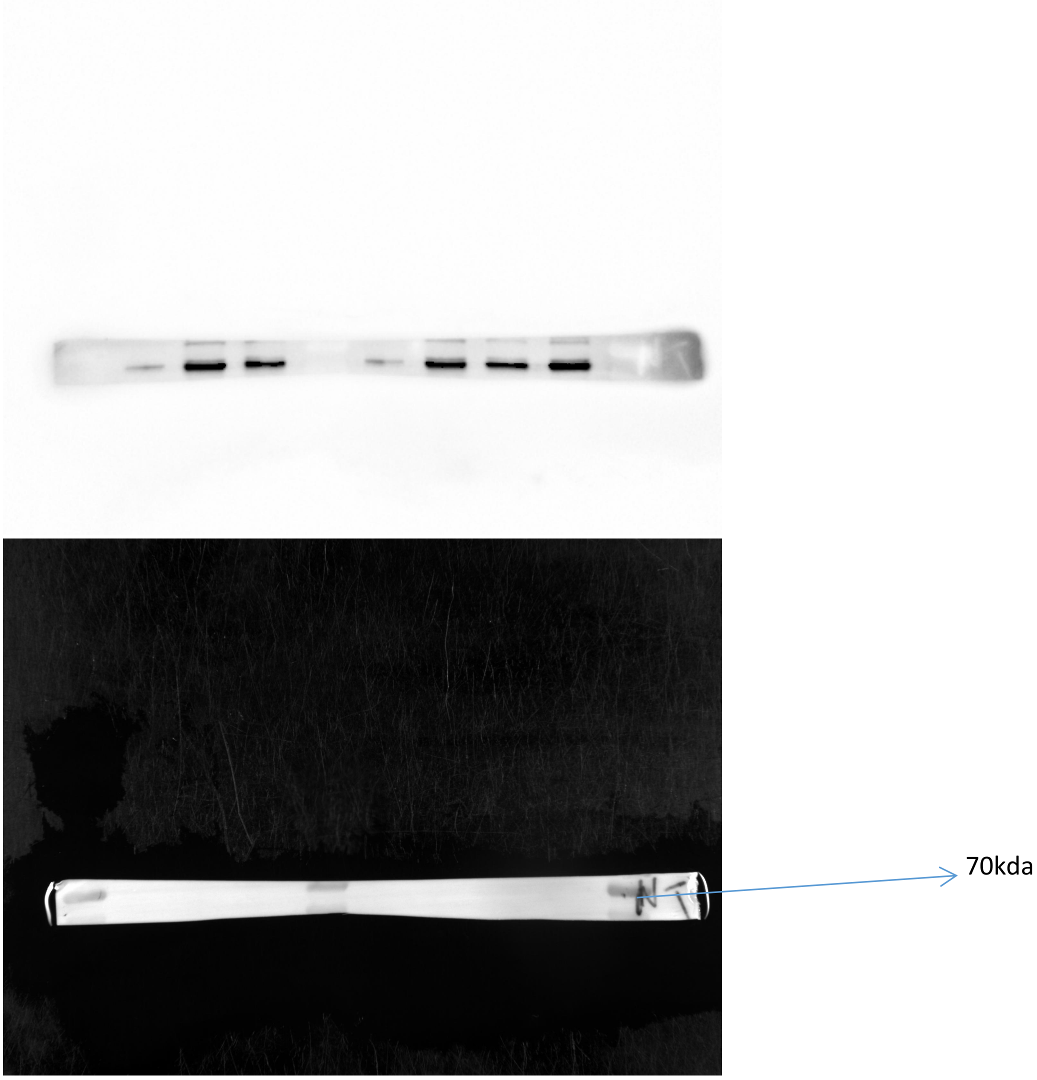

Figure6：p-NF-kB p65(65KDa)

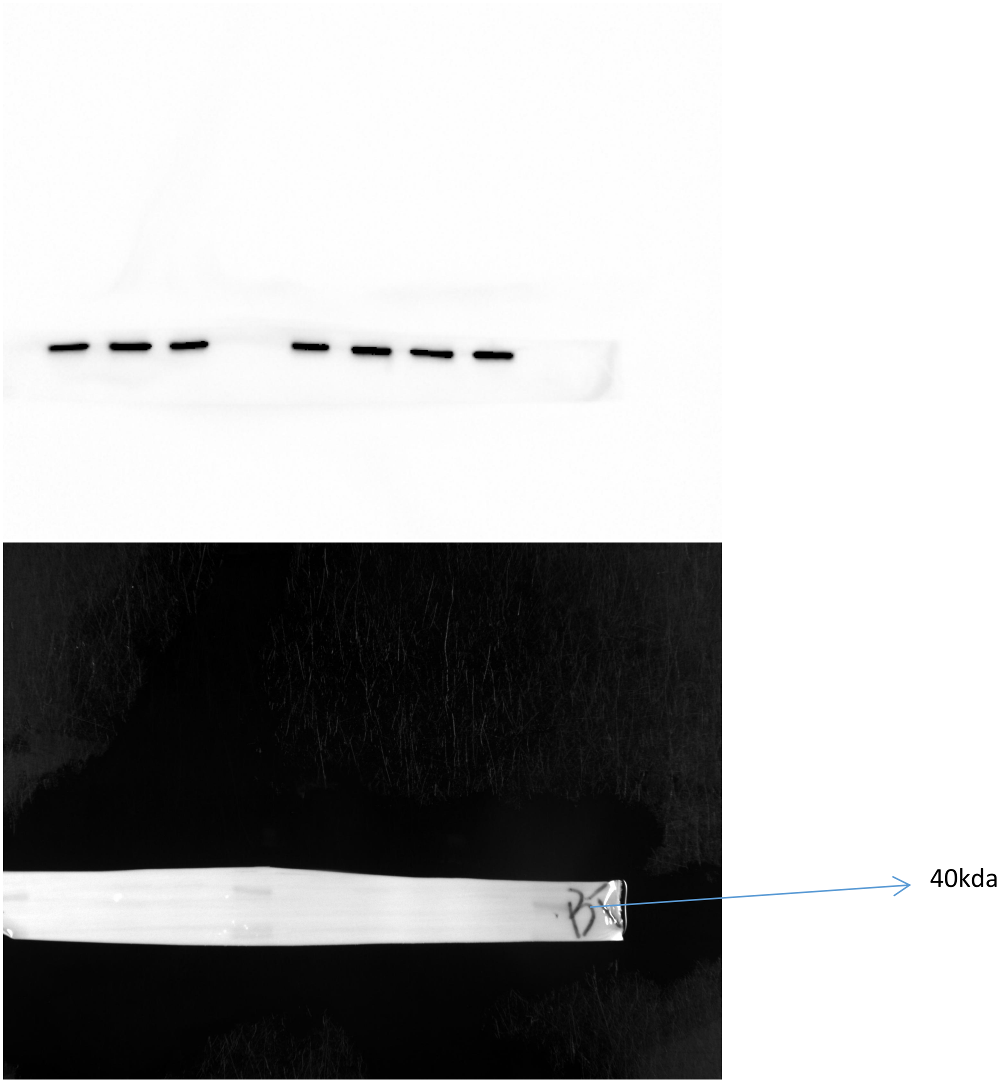

Figure6：p-NF-KB p65-actin(42.5KDa)

Maker: Beyotime, P0075 and Thermo Fisher, 26616.
